# Spatial memory shapes density dependence in population dynamics

**DOI:** 10.1098/rspb.2017.1411

**Published:** 2017-11-22

**Authors:** Louise Riotte-Lambert, Simon Benhamou, Christophe Bonenfant, Simon Chamaillé-Jammes

**Affiliations:** 1 Centre d'Ecologie Fonctionnelle et Evolutive, CNRS, Université de Montpellier, 1919 Route de Mende, 34293 Montpellier Cedex 5, France; 2 Institute of Biodiversity, Animal Health and Comparative Medicine, College of Medical, Veterinary, and Life Sciences, University of Glasgow, Graham Kerr Building, Glasgow G12 8QQ, UK; 3 Laboratoire de Biométrie et Biologie Évolutive, CNRS, Université Claude Bernard Lyon 1, – Bat. Grégor Mendel, 43 bd du 11 novembre 1918, 69622 Villeurbanne cedex, France

**Keywords:** memory, movement, population dynamics, foraging, space use

## Abstract

Most population dynamics studies assume that individuals use space uniformly, and thus mix well spatially. In numerous species, however, individuals do not move randomly, but use spatial memory to visit renewable resource patches repeatedly. To understand the extent to which memory-based foraging movement may affect density-dependent population dynamics through its impact on competition, we developed a spatially explicit, individual-based movement model where reproduction and death are functions of foraging efficiency. We compared the dynamics of populations of with- and without-memory individuals. We showed that memory-based movement leads to a higher population size at equilibrium, to a higher depletion of the environment, to a marked discrepancy between the global (i.e. measured at the population level) and local (i.e. measured at the individual level) intensities of competition, and to a nonlinear density dependence. These results call for a deeper investigation of the impact of individual movement strategies and cognitive abilities on population dynamics.

## 1. Introduction

Density dependence is a major feature of population dynamics and its pervasiveness in wild populations has been demonstrated repeatedly (meta-analysis in [1]). As it determines population growth and variability of population size through intra-specific competition, it affects the resilience of populations and their probability to go extinct [2]. Thus, a good understanding of processes by which density dependence occurs is required to predict population dynamics in both fundamental and applied research [3,4]. Following the early publication of the logistic population growth model [5], many other phenomenological models (e.g. Beverton–Holt [6]; Ricker [7,8]; Gompertz [9]; theta-logistic [10]; density-threshold [11]) were proposed to describe the relationship between the population growth rate and the overall population density. This diversity of models partly stemmed from the need to describe the wide variety of shapes of density dependence curves revealed by empirical studies (review in [12]; meta-analysis in [13]).

The different shapes of density dependence observed in empirical data have often been assumed to emerge from the way the demographic parameters of age- or stage-structured populations are functions of the density and combine to determine population growth rates [14,15]. For instance, in large mammals, juvenile survival starts decreasing at lower densities than adult survival does [16,17], resulting in a dropping curve rather than a linear relationship between the *per capita* growth rate and population density [18]. Most models of density dependence also assume that (i) the environment defines a (possibly time-varying) potential for competition (the so-called ‘carrying capacity') around which the population density will fluctuate more or less widely, depending on the shape of density dependence, and (ii) the overall density, as measured by the ratio of the total population size over the area accessible to the animals, is a relevant index of the intensity of conspecific competition [14,19,20] (reviewed in [21]). When considering a population of mobile animals, this latter assumption implicitly means that individuals mix well spatially, i.e. that any individual encounters others with the same rate, and therefore that the intensity of conspecific competition experienced by individuals is homogeneous over the population spatial range [21,22].

Behavioural interference can break these assumptions. For instance, population dynamics can be impacted by deleterious interactions and spatial segregation promoted by non-parental female infanticide [23], reproductive suppression [24], and territoriality [25–27]. Such behaviours involve agonistic interactions that can negatively impact population growth rates [28], in a density- and environment-dependent way [29]. However, animals can reduce competition pressure without aggressive behaviour simply by using personal information when their environment is partly predictable. The ability to memorize the location of high-quality patches of depletable but renewable resources, by using a reference (i.e. long-term) memory, and to keep track of the time elapsed since the last visit to a patch, by using a working (i.e. short-term) memory, has been hypothesized to be a major determinant of resource use efficiency [30,31]. Memory-based foraging improves foraging efficiency by decreasing the time spent travelling by focusing on the best known patches and by improving the timing of revisits to these patches [32–34]. Recently, it was shown that memory-based foraging leads individuals to display recursive movement patterns (home ranges), and to segregate as they passively avoid areas that seem of lower quality because they are depleted by competitors [33]. Such spatial effects of memory-based foraging thus invalidate common implicit assumptions of density-dependence models, such as perfect spatial mixing of individuals. Moreover, the better foraging efficiency induced by spatial memory-based foraging should increase demographic performance, unless it is counterbalanced by more resource depletion.

Memory-based foraging movement is therefore likely to affect how population density translates into competition intensity and thereby the shape of density dependence and ultimately the dynamics of populations. To the best of our knowledge, no study has tried to clarify the expected link between memory-driven resource use and population dynamics. Here, we addressed this long-overdue challenge [21] by exploring how memory-based foraging may shape resource and population dynamics. We developed an individual-based model integrating simple movement rules and their consequences on life histories to compare the population dynamics of populations of with- and without-memory individuals separately, to answer three key questions:

(1) As memory use improves the foraging efficiency of individuals, does it result in a larger carrying capacity of the environment and/or in stronger resource depletion for a given population size?
(2) As memory use leads to restricted space use and to some degree of spatial segregation, does it lead to a discrepancy between the actual intensity of competition experienced by a given individual and the one expected when using the overall population density (the ratio of the total population size over the area accessible to the animals) as an index of competition?
(3) As memory use enables individuals to dynamically adapt their exploitation of resources to local conditions and to segregate, but probably only up to some population density [33], can it lead to a nonlinear density dependence of the population growth rate?

## 2. Modelling

### (a) Environmental make-up and resource dynamics

We extended a mechanistic movement model initially developed in [33] to investigate how spatial memory affects the way animals share space and resources. The environment is a 100 × 100 u^2^ (where u is an arbitrary length unit) continuous-space square with reflective boundaries. It contains *N_p_* = 400 resource patches that can each contain the same maximum amount *Q*_max_ of resources. Patch centres are randomly distributed in space (i.e. the *x*, *y* coordinates of any patch centre are drawn at random from a uniform distribution, independently of each other and independently of the locations of other patches). This results in a Poisson distribution of the number of patches per unit area. We checked, however, that our results were not affected by spatial clustering in the distribution of patches (electronic supplementary material, S1). We deliberately ignored intra-patch movements, by assuming that they were performed in the same way by animals with or without memory. Indeed, at this small spatial scale, a reference memory should become irrelevant, items within patches being assumed to be directly detected at a distance, or found using area-concentrated search, which requires either no memory at all, or only an ephemeral spatial working memory [35]. Consequently, all patches were simulated as points in space. This is a common assumption made in movement models focusing on larger-scale processes and memory-based movements [36].

When an individual reaches a patch, it consumes almost all available resources in one time unit, lowering their amount to *Q*_max_/1 000. This is a simplifying assumption, as an individual could leave a patch after consuming only a fraction of the resources available, for instance because of trade-offs between foraging longer and predation risk or other nutritional needs. However, complete patch depletion in one visit has been reported in some real systems (e.g. nectarivores [37]), and it can be assumed here because the important point in our study is that energy is a limiting factor. The amount of resources within any patch *p* is renewed over discrete time steps according to a logistic growth function with the renewal rate set to 0.03 in all simulations: *P*[*p*]*_t_*_+1_ = *P*[*p*]*_t_* + 0.03 *P*[*p*]*_t_*(1–*P*[*p*]*_t_*), where *P*[*p*]*_t_* is the proportion (with respect to maximum content *Q*_max_) of resources that the patch *p* contains at time *t*.

### (b) Movement strategies

All individuals move independently of each other, with a constant speed of 1 u per time step whatever their movement strategy, and detect a patch when they come within 2 u of it. We considered two types of populations separately, one composed of memoryless individuals, and the other of with-memory individuals. Memoryless individuals simply search for food by performing a correlated random walk characterized by unit step lengths and a zero-centred wrapped normal distribution of turns with a mean cosine equal to 0.8, but ignore patches visited in the previous 10 time steps to prevent systematic backtracking to the same close patches. With-memory individuals store the location of any detected patch and its expected quality (see below) in a reference memory. If the patch is not revisited before *T*_R_ = 1 000 time steps, this information (patch location and expected quality) is forgotten. These individuals also rely on a working memory that retains for a duration *T*_W_ = 200 time steps that they have recently visited the patch, preventing them from returning to it until a time longer than *T*_W_ has elapsed (*T*_W_ = 200 allows for a minimal replenishment of the patch at 30% if no other individual visits it in the meantime). When a with-memory individual visits a patch *p* for the first time (or revisits it after more than *T*_R_ time steps), the patch quality it expects, *Q*[*p*]*_t_*, is set to the quantity of food it finds at this time: *Q*[*p*]*_t_* = *P*[*p*]*_t_ Q*_max_. When the individual revisits this patch later at time *t* + *τ* with *T*_W_ < *τ* < *T*_R_, the memory time counter is reset to 0 and the expected patch quality is updated to the arithmetic mean between the amount of resources expected, which reflects its previous experience, and the amount of resources actually found, which reflects its current experience: *Q*[*p*]*_t_*_+*τ*_ = (*Q*[*p*]*_t_* + *P*[*p*]*_t_*_+*τ*_ *Q*_max_)/2, and remains at this value until it revisits the patch. This simple rule allows individuals to adapt the timing of patch revisits to patch renewal dynamics by trial and error [33]. The behaviour of an individual leaving a patch depends on the attractiveness (ratio *Q*[*p*]/*D*[*p*] where *D*[*p*] is the current distance to patch *p*) of every known patch that was exploited at least *T*_W_ time steps ago. If at least one patch presents an attractiveness higher than a threshold set to 0.01 for all simulations, the individual chooses the patch with the highest attractiveness and travels to it along a straight line. Otherwise, it will rely on the same random search as memoryless individuals until an unknown patch is perceived or a known patch's attractiveness becomes larger than 0.01.

### (c) Linking foraging movement behaviour to survival and reproduction

Hereafter, we express energy levels, gains, and costs with respect to *Q*_max_, the maximum amount of resources a patch can contain (i.e. *Q*_max_ is considered the energy unit and can therefore be removed from these expressions). Survival and reproduction of an individual *i* depend on its energy level at time *t*, *E*[*i*]*_t_*. At each time step, it either decreases by 0.05 (corresponding to the energetic cost of movement per unit time) for an individual that is travelling, or increases by *P*[*p*]*_t_* − 0.001 for an individual that is exploiting the resource patch *p*. The individual dies when *E*[*i*]*_t_* reaches 0, and asexually reproduces when *E*[*i*]*_t_* reaches 500, but reproduction costs a random value uniformly drawn between 240 and 260. The offspring is born at the location where its parent's state reached 500 and gets a random initial energy level uniformly drawn between 90 and 110. All individuals are born naive, but while the offspring of a memoryless individual will remain naive for its lifetime, the offspring of a with-memory individual will immediately start to learn patch locations and qualities as explained above.

### (d) Local versus global intensity of competition

We ran 1 000 simulations for 200 000 time steps for each movement strategy separately. A new environment was drawn for every simulation run, which started with the introduction of a single naive individual at a random location in the landscape, with *E*[*i*]_0_ randomly drawn between 240 and 260. We deleted simulations for which the population went extinct. This never happened for populations of with-memory individuals, and happened in less than 4% of simulations of populations of memoryless individuals. We divided each simulation run into 5 000 time-step-long contiguous windows at the beginning and end of which we recorded the number of individuals alive and the resource level in the environment expressed as the mean standing crop (mean current amount of resources) in a patch. For each individual *i* alive at the beginning of a time window *j*, we recorded the number of offspring it produced during this window, and, if it was still alive at the end of the window, we computed a measure of the local intensity of competition *I*_loc_[*i*]*_j_* it experienced during this window as: *I*_loc_[*i*]*_j_* = *N_v_*[*i*]*_k≠i_*_,*j*_/(*N_v_*[*i*]*_i,j_ N_i,j_*), where *N_i,j_* is the number of patches visited at least once by individual *i* during the time window *j*, and *N_v_*[*i*]*_i,j_* and *N_v_*[*i*]*_k≠i_*_,*j*_ are the total numbers of times any of the *N_i,j_* patches was visited by individual *i* and by any individual other than *i*, respectively, during this same time window *j*. For example, if these *N_i,j_* patches were equally exploited by *k* individuals including individual *i*, one gets *I*_loc_[*i*]*_j_* = (*k–*1)/*N_i,j_*, which reduces to 0 if *i* was the only individual (*k* = 1) to exploit them.

For each type of movement strategy, we investigated the relationship between the local and global competition intensities. The local intensity of competition corresponds to the average, over all individuals, of *I*_loc_[*i*]*_j_*. The global intensity of competition was computed as the ratio (*n* − 1)/*N*_*p*_, where *n* is the population size (*n* − 1 thus corresponds to the number of competitors faced by any given focal individual *i*) present in the environment at the beginning of the time window *j* and *N*_*p*_ is the number of patches. As *N*_*p*_ was kept constant (*N*_*p*_ = 400), the global competition intensity is thus proportional to the traditional measure of population density used in population dynamics studies, expressed as the number of individuals (the total population size) divided by the area accessible to the individuals.

### (e) Characterization of density dependence at the population level

To determine the shape of density dependence, we looked at the mean *per capita* growth rate as a function of the population size. We computed the *per capita* growth rate of the populations as *r* = ln (*n_t_*_+1_/*n_t_*) (which is the discrete time counterpart of the continuous time expression *r* = d*n*/(*n*d*t*)), where *n_t_* is the population size at time *t*. We averaged the various values of *r* obtained per simulation and per population size to retain only one point per simulation and population size. We first fitted the Ricker model [7,8] as our baseline linear model of density dependence. As the growth rates of populations of with-memory individuals appeared to decrease nonlinearly with population size (see the Results section), we then investigated nonlinearity by fitting the Beverton–Holt model [6,38], the theta-logistic model, where the shape parameter *θ* was estimated from the data, and a second-order polynomial regression. The Beverton–Holt model is not linear (although it may approach linearity for small intrinsic growth rates) and is a discrete-time analogue of the logistic growth in continuous time [38]. The theta-logistic model is one of the most widely used phenomenological models to describe nonlinear density dependence [13,38]. It corresponds to *r* = *r*_max_ (1 − (*n*/*K*)*^θ^*), where *r*_max_ is the intrinsic growth rate (i.e. when the population is not limited by density dependence), *K* is the carrying capacity and *θ* describes the curvature of the relationship [39]. For *θ* = 1, the theta-logistic model reduces to the Ricker model [7,8]. Because the nonlinearity in populations of with-memory individuals visually appeared to be mainly caused by an abrupt break point, we also fitted a piecewise second-order polynomial regression with a break point estimated from the data. We compared the fits of those models for both types of population using the Akaike information criterion (AIC, [40]).

### (f) Sensitivity analyses

We ran additional simulations (100 for each parameter setting) to investigate the sensitivity of the carrying capacity and of the occurrence, location, and abruptness of the break point in the density-dependence curves of populations of with-memory individuals to variations in the values of key parameters. We varied the duration of the working memory *T*_W_ between 200 (the default value) and 1 000, and the duration of the reference memory *T*_R_ between *T*_W_ + 100 and 1 200 (as *T*_R_ needs to be higher than *T*_W_; see [33] for details; electronic supplementary material, S2). We also simulated populations with *T*_W_ = 100, but all went extinct. Setting all other parameters to default values, we investigated the effect of the attractiveness threshold used by individuals to determine valuable patches (electronic supplementary material, S3), and of the energetic cost of movement (electronic supplementary material, S4), by halving or doubling their default values (0.01 and 0.05, respectively). We varied the difference between the energy threshold that an individual must reach to reproduce and the value to which its state is lowered following a reproduction event by modifying the former such that the default difference (250) was halved or doubled (electronic supplementary material, S5).

All simulations and analyses were performed using Julia v. 0.3.9 [41] and R [42], respectively.

## 3. Results

### (a) Memory use entails a higher carrying capacity

**Figure 1.**
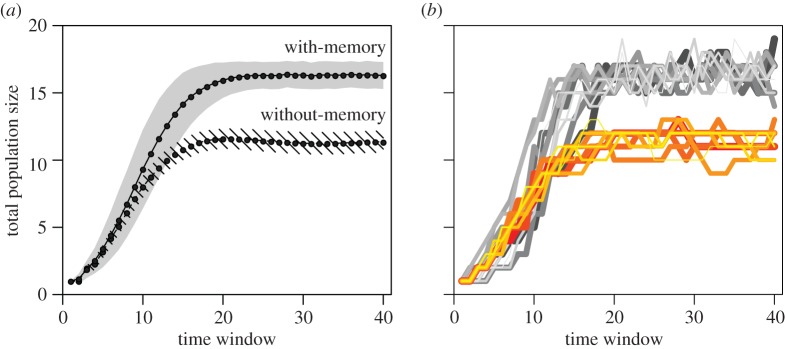
Total population size as a function of the time elapsed since the population's founding (*n* = 1), measured at the beginning of each time window lasting 5 000 time steps each. (*a*) Mean ± s.d. (over 1 000 simulations for each population type), for populations of with- and without-memory individuals (plain and dashed lines, respectively). (*b*) Examples of individual population growth curves. Ten examples are shown for each population type (grey: with-memory; orange: without-memory). Different populations are represented with different line widths for ease of visualization.

The carrying capacity of the environment for populations of with-memory individuals is 45% larger than that of populations of memoryless individuals (mean ± s.d.: 16.3 ± 0.3 versus 11.3 ± 0.5; figure 1*a*). For each type of population, the value of the carrying capacity of the environment is only slightly affected by changes in the values of the parameters we tested except the cost of movement (electronic supplementary material, S2–S5). Most populations of without- (100%) or with-memory (76%) individuals go extinct when the cost of movement is set to twice the default value, whereas the carrying capacities are increased when the cost of movement is halved and become almost equal between the two types of populations (electronic supplementary material, S4).

### (b) Memory use entails a stronger environmental depletion

**Figure 2.**
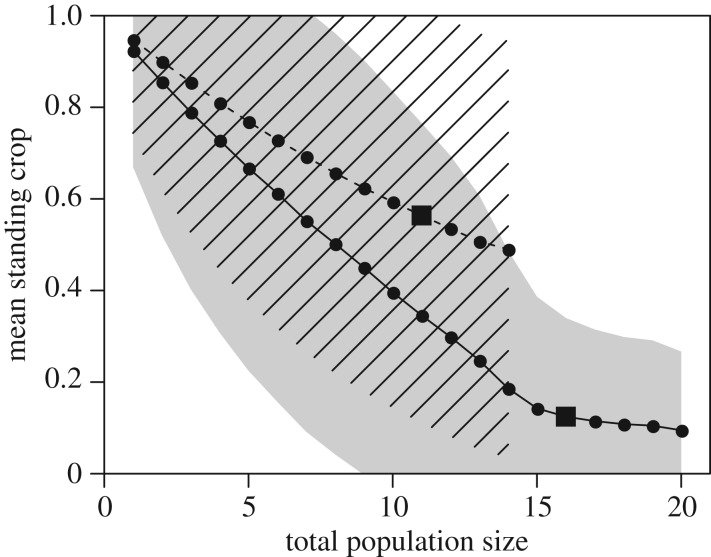
Mean±s.d. standing crop in the patches (expressed as a fraction of the maximum patch resource capacity *Q*_max_) at the end of each time window, as a function of the total population size, for populations of memoryless (dashed line and zebra area) versus with-memory individuals (plain line and grey area). The variation shown is the mean standard deviation between patches within simulations. The squares represent the mean standing crop at the carrying capacity for both types of populations.

The standing crop, i.e. the mean amount of resources present in the patches at any time, is on average smaller in populations of with- than in populations of without-memory individuals, and the difference increases with the population size (figure 2). In particular, the mean standing crop in populations of with-memory individuals is 78% smaller than the one observed in populations of memoryless individuals when these populations are at their equilibrium. A large variability of the standing crop between patches within simulations occurs, due to the highly dynamic nature of our system, where patches regrow progressively with time but are depleted episodically.

### (c) Memory use entails a difference between local and global levels of competition

**Figure 3.**
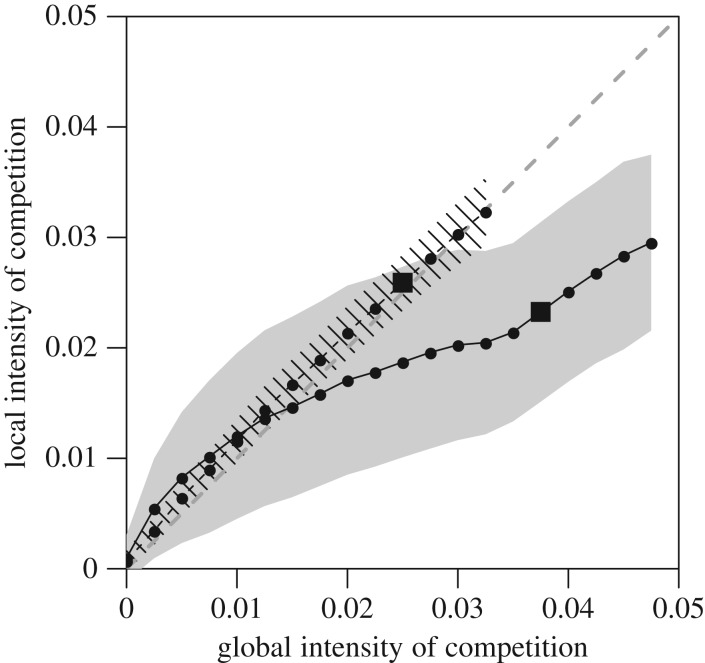
Mean ± s.d. local intensity of competition as a function of the global intensity of competition (aka population density), for populations of memoryless (dashed line and zebra area) and with-memory (plain line and grey area) individuals. The dark grey dashed line represents the isometric relationship for comparative purposes. The s.d. shown is the mean s.d. between individuals within simulations. The squares represent the global intensity of competition at the carrying capacity for both types of populations.

In populations of without-memory individuals, the local (i.e. individually experienced) and global intensities of competition are always similar, whereas in populations of with-memory individuals, this holds true only at low density (less than 0.025; figure 3). At higher density, the local intensity of competition increases much slower than the global one. It is also worth noting that there is a higher level of heterogeneity of local intensities of competition in populations of with- than in populations of without-memory individuals.

### (d) Memory use entails a nonlinear density dependence

**Figure 4.**
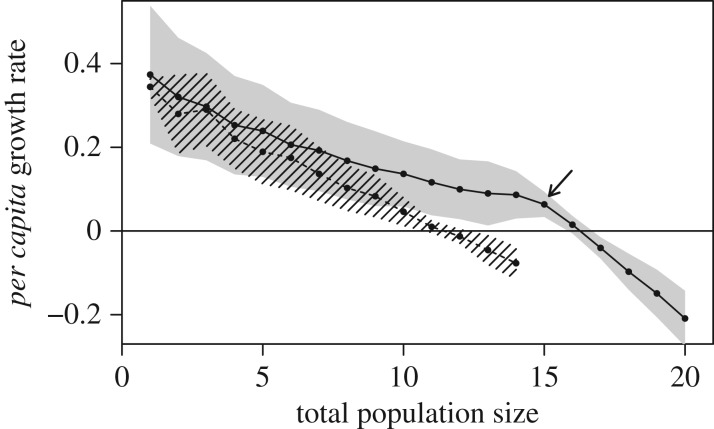
Mean ± s.d. (between simulations) *per capita* growth rate as a function of total population size, for populations of memoryless (dashed line and zebra area) and with-memory individuals (plain line and grey area). Populations made of more than 14 memoryless individuals were never observed. The arrow indicates the estimated location of the break point.

The shape of density dependence differs between populations of with- and without-memory individuals (figure 4). The population growth rate decreases with the size of the population almost linearly for memoryless individuals but clearly nonlinearly for with-memory individuals. This nonlinearity results from a sudden increase in the strength of density dependence, i.e. a break point. As a consequence, a piecewise second-order polynomial regression describes the data much better than a simple second-order polynomial regression, or the Ricker, Beverton–Holt, or theta-logistic models (all ΔAIC > 1 000; electronic supplementary material, S6 for a graphical comparison of the model fits and the parameter estimates). With the parameters set to default values, the break point is estimated to occur at a population size of about *n* = 15, and at this population size, the slope of density dependence is multiplied by 10.8 (calculated as the ratio of the slopes of the fitted model right before and right after the break point). For populations of memoryless individuals, the fitting procedure of the piecewise second-order polynomial regression does not converge, and the best fitting model is the theta-logistic model with shape parameter *θ* close to 1 (*θ* = 0.88; ΔAIC: 45 with the Ricker (i.e. linear) model, 22 with the Beverton–Holt model, and 11 with the second-order polynomial regression). Overall, all models correspond to a similar, linear, or near-linear, relationship (electronic supplementary material, figure S15).

For populations smaller than 15 individuals (i.e. before the break point that occurs for populations of with-memory individuals), density dependence is stronger for without- than for with-memory populations, as indicated by the steeper slope of the density-dependence curve for the former than for the latter (figure 4). After the break point, with-memory populations experience the highest levels of density dependence (figure 4).

The sensitivity analyses revealed that the occurrence and location of the break point for populations of with-memory individuals are little affected by changes in the length of working or reference memories (electronic supplementary material, S2). However, the magnitude of the change in the slope of the density dependence at the break point decreases when *T*_W_ increases (electronic supplementary material, figure S8), leading to almost linear density-dependence curves for *T*_W_ ≥ 500 (electronic supplementary material, figure S9). The occurrence of the break point and its location for populations of with-memory individuals are little affected by changes in the difference between the energy threshold that an individual must reach to reproduce and the value to which its state is lowered following a reproduction event. In both cases, the change of slope before and after the break point is abrupt (electronic supplementary material, S5). When the threshold used by individuals to select valuable patches is doubled, there is no break point (electronic supplementary material, S3), and when the energetic cost of locomotion is halved, density-dependence curves of with- and without-memory populations become very similar (electronic supplementary material, S4).

## 4. Discussion

Memory-based foraging is very common among mobile organisms, at least in vertebrates [31]. It affects how animals use and share resources, resulting in recursive movement patterns (home ranges) and spatial segregation between individuals [33]. By linking foraging behaviour with life histories, our study demonstrates, for the first time, that the use of memory is at variance with the implicit assumption of perfect spatial mixing of individuals made in classical models of density-dependent population dynamics and can have significant consequences for the interpretation of population dynamics patterns.

Differences in population growth rates and how these rates relate to density are most commonly attributed to environmental resource availability, age structure of populations, and/or interactions between life-history traits [13–15,43,44]. For instance, in large mammals, the reduction in the population growth rate is expected to become stronger as the population size increases, leading to a dropping curve, because the life-history traits with the greatest impact on population growth (e.g. adult survival) become affected by population density only at larger population sizes [2]. In our model, however, both with- and without-memory individuals face the same initial environment and have life histories driven similarly by their energy state. The differences in the shapes of density dependence between our two population types therefore only emerge because of the ability/inability of individuals to memorize the locations and quality of resources patches. As expected, populations of memoryless individuals, which mix well spatially because of the randomness of their movements, experience scramble competition and display near-linear density dependence [45]. By contrast, populations of with-memory individuals display nonlinear density dependence that could not be adequately described by any of the classical models of density dependence we fitted. This result is, however, sensitive to the cost of movement and to the working memory. With a long working memory, the density-dependence curve approaches linearity (electronic supplementary material, S2), probably because a long working memory leads to larger and more overlapping home ranges [33]. With a small cost of movement, both types of populations experience a high carrying capacity and a similar density-dependence shape (electronic supplementary material, S4). This convergence occurs because at the very high population density reached in this situation, with-memory individuals do not tend to perform home range behaviour (and thereby to spatially segregate), and therefore forage with an efficiency similar to that of memoryless individuals (see electronic supplementary material, S4 for examples). By contrast, with a high cost of movement, almost all populations go extinct.

Despite its flexibility and its wide use in population dynamics studies [19,20], the theta-logistic model poorly fits the shape of the density dependence we obtained for populations of with-memory individuals. More generally, all the standard models of density dependence we considered poorly fitted this shape, as they are unable to account for the break point occurring at a population size lower than the carrying capacity and beyond which density dependence sharply strengthens. At this population size, the mean standing crop reaches its asymptotic lowest level. Interestingly, this break point occurs at a population size that is very close or equivalent to the maximum population size reached by populations of without-memory individuals. Thus, beyond this population size, no additional individual without memory can survive, because the environment becomes too depleted for them. As with-memory individuals give birth to naive offspring, very few of them can compete for resources with older individuals and thus survive in such a severely crowded and depleted environment. Consequently, the *per capita* growth rate drops sharply beyond the break point population size.

Most studies of density dependence use the total population size or the mean density over the study area to index the intensity of competition for food resources within the population (reviewed in [21]). The only demographic studies that quantified local population densities at a relevant scale and that accounted for local heterogeneities focused on non-mobile organisms [46], territorial animals [47], or animals living in environments that are heterogeneous at a large spatial scale [48–50] (but see [51]). In our study, the environment was heterogeneous at very small scale (smaller than the smallest scale of individuals' movement considered here) but homogeneous at larger scale (equal or larger than inter-patch movements). Even so, memory-based movement led to heterogeneous individually experienced intensities of competition and, for a wide range of population sizes, to much smaller than expected mean intensities of individually experienced competition. This highlights the importance of the spatial scale considered when measuring the intensity of competition for studying its influence on demographic rates: the total population size may not accurately reflect the intensity of competition experienced at the individual level, even when the environment is homogeneous.

Our conclusions obtained in an environment where resources are aggregated in randomly distributed patches (single level of heterogeneity) remain valid in an environment where the patches themselves are clustered in super-patches (two levels of heterogeneity; electronic supplementary material, S1). This occurs because memoryless individuals remain intrinsically unable to establish home ranges, and therefore continue to form well-mixed populations (no spatial segregation), whereas with-memory individuals can efficiently navigate within and between super-patches. This suggests that our results are likely to apply to a wide array of situations. Therefore, we trust that whenever studied individuals commonly perform recursive movements, a likely indicator of the use of memory [31], the assumption of well-mixing of individuals within a population is also probably invalid, and further investigations of the link between space use and demography are necessary. Beyond this, our study highlights that a shift from pattern-oriented (i.e. phenomenological) studies to process-oriented studies [52] seems critical to limit the risk of false inference about the underlying biological processes generating specific shapes of density dependence.

Our model also leads to carrying capacities that are larger for populations of with- than of without-memory individuals, even though memory users deplete their environment more than memoryless individuals. This result is very robust to changes in model parameters, and clearly demonstrates that the carrying capacity does not just result from the interaction between the environment and species' demographic traits, but also depends on the way individuals exploit their environment. With-memory individuals probably maintain high levels of resource intakes in highly depleted environments because (i) they time their revisits to known patches better, thereby increasing the amount of food found at each visit, and (ii) they limit the time spent travelling, thereby decreasing the overall cost of visiting a patch [33]. To the best of our knowledge, no study has previously investigated the effect of foraging strategies on the carrying capacity, except for territorial animals [21]. Our result goes against the historical but recently challenged assumption that a gain in fitness is compensated for by a strong negative density dependence due to environment deterioration, e.g. that the selection for a behavioural trait will not change the carrying capacity [53] (reviewed and discussed in [54–56]). For this reason, but also for mathematical convenience, many evolutionary models set population sizes to a constant value [57,58] (but see [59]). Our results question this practice. The evolutionary emergence and improvement of memory-based foraging behaviour should be selected positively in environments that are at least partially predictable, as it brings important fitness benefits [31,33,60]. Our work suggests that the selection for better memory capacities should alter consumer-resource dynamics by increasing resource depletion while increasing the carrying capacity of the environment. In turn, these effects should reinforce the evolutionary processes selecting for improved cognitive capacities, because the relative advantage of having a better memory increases with the depletion of the environment. Our results have thus connections with the fast-growing field of eco-evolutionary dynamics [61] and urge investigation of how the strength of natural selection can be impacted by the selection of cognitive traits that modify carrying capacity [56].

## Supplementary Material

Sensitivity analyses and details on the model fits

## Data accessibility

The Julia codes supporting this article are freely available on Figshare [62].
