# Supplementary material for: Spatial memory shapes density dependence in population dynamics
Source: Proc Biol Sci. 2017 Nov 22;284(1867):20171411. doi: 10.1098/rspb.2017.1411 (PMC5719166; doi:10.1098/rspb.2017.1411)
Supplement: Sensitivity analyses and details on the model fits [file rspb20171411supp1.pdf]

Supplementary materials for the article:

Spatial memory shapes density-dependence in population dynamics

Proceedings of the Royal Society B

Louise Riotte-Lambert[a,b], Simon Benhamou[a], Christophe Bonenfant[c], Simon Chamaillé-Jammes[a]

[a] Centre d'Ecologie Fonctionnelle et Evolutive, UMR 5175, CNRS - Université de Montpellier, 1919 Route de Mende, 34293 Montpellier Cedex 5, France.
[b] Institute of Biodiversity, Animal Health and Comparative Medicine, College of Medical, Veterinary, and Life Sciences, Graham Kerr Building, University of Glasgow, Glasgow G12 8QQ United Kingdom
[c] Laboratoire de Biométrie et Biologie Évolutive, UMR 5558, CNRS, Université Claude Bernard Lyon 1, – Bat. Grégor Mendel, 43 bd du 11 novembre 1918, 69622 Villeurbanne cedex, France

Corresponding author:

Louise Riotte-Lambert: louise.riotte.lambert@gmail.com

doi: 10.1098/rspb.2017.1411

Contents:

Supp Info 1: *Effect of the spatial clustering of resource patches………………………………………… 2*

Supp Info 2: *Sensitivity of the carrying capacity and of the shape of the density-dependence of populations of with-memory individuals to the durations of working (T_W) and reference (T_R) memories…………………………………………………………………………………………….. 7*

Supp Info 3: *Sensitivity of the carrying capacity and the shape of the density-dependence of populations of with-memory individuals to the attractiveness threshold used by individuals when determining valuable patches………………………………………………………………………… 36*

Supp Info 4: *Sensitivity of the carrying capacity and of the shape of the density-dependence to the energetic cost of movement………………………………………………………………………… 37*

Supp Info 5: *Sensitivity of the carrying capacity and the shape of the density-dependence to the value of the energetic threshold that an individual must reach to reproduce………………………………… 39*

Supp Info 6: *Comparison of three phenomenological models of density-dependence fitted on r-N curves…………………………………………………………………………………………………… 40*

Supporting Information 1

Effect of the spatial clustering of resource patches

We simulated an environment with patches aggregated in super-patches following Benhamou (1992)[1]. The same number of identical resource patches as in the main analyses (N_p = 400) were distributed among 20 clusters, with a within-cluster patch density of 0.15 (Fig. S1).

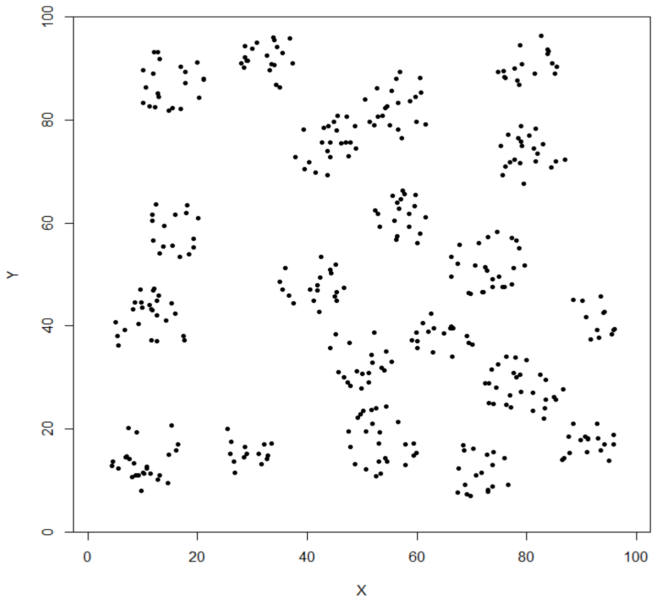

Fig. S1. Example of an environment with patches aggregated in super-patches.

Running the model on this spatially clustered environment did not qualitatively change our results: the carrying capacity of populations of memory users was still much higher than that of non-memory users, and both carrying capacities were close to that in the non-clustered case (Fig. S2), and memory use led to heavy environmental depletion (Fig S3), to smaller local intensities of competition for high global population densities (Fig S4), and to nonlinear density-dependence (Fig S5-6).

[1] Benhamou (1992). Efficiency of area-concentrated searching behaviour in a continuous patchy environment. *Journal of Theoretical Biology*. 159:67-81.

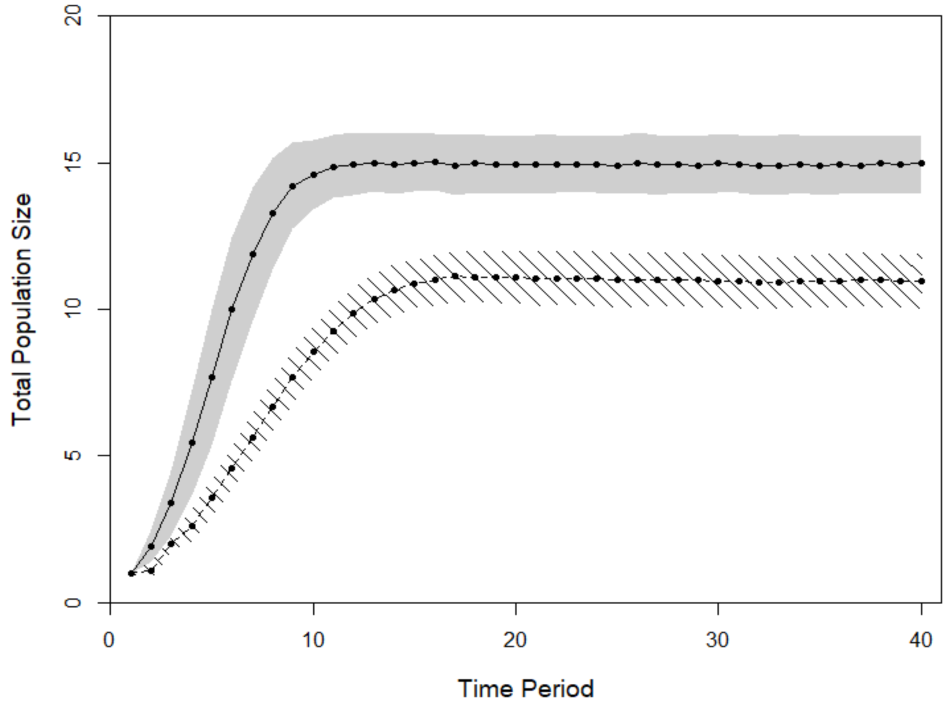

Fig S2: Total population size (mean + SD) as a function of the time elapsed since the population's founding (*n*=1), measured at the beginning of each time window lasting 5,000 time steps each, for populations of with-memory (plain line and grey area) and without-memory individuals (dashed line and zebra area)

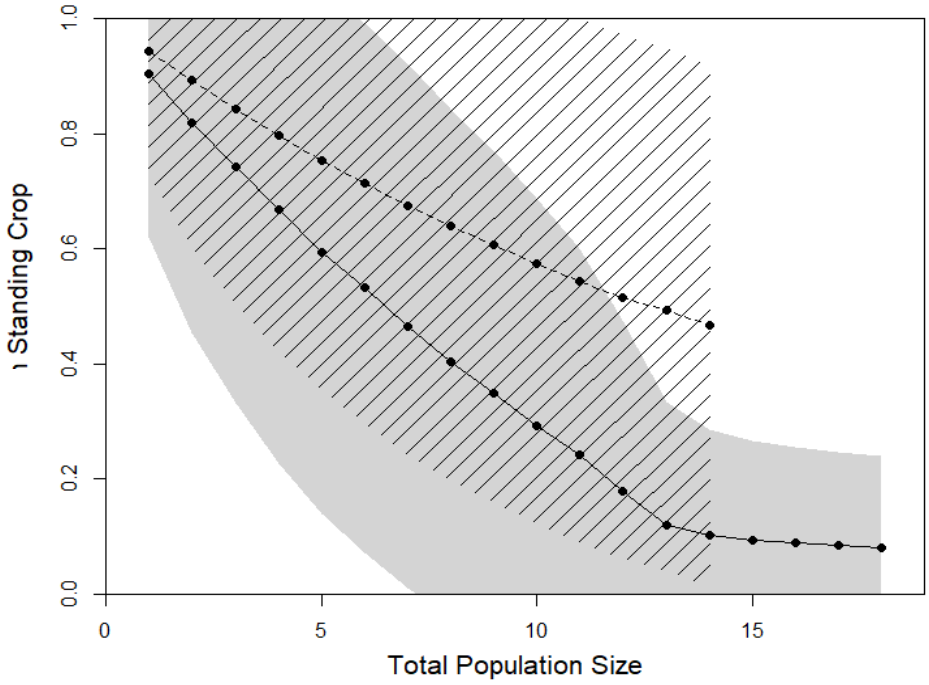

Fig. S3. Standing crop (mean + SD) in the patches at the end of each time window, as a function of the total population size, for populations of memoryless (dashed line and zebra area) and with-memory individuals (plain line and grey area). The variation shown is the mean standard deviation between patches within simulations.

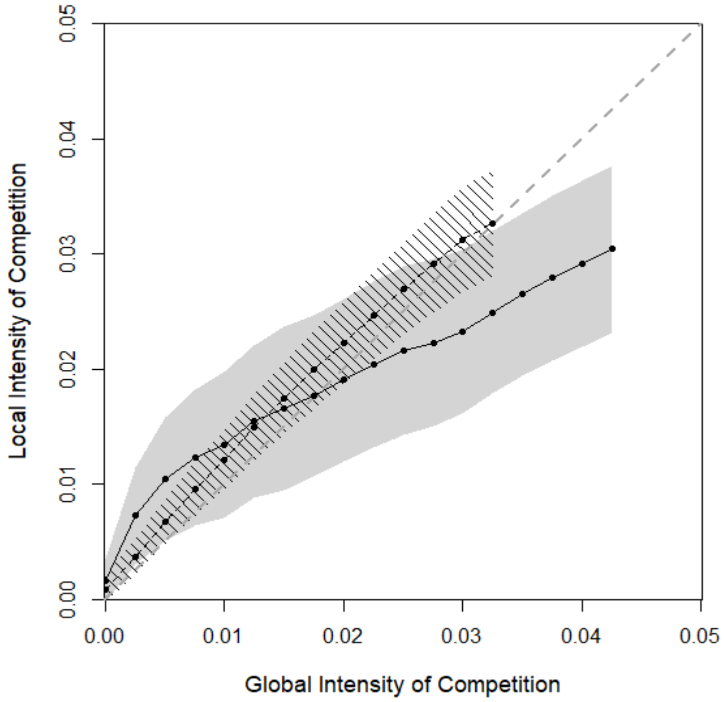

Fig S4. Mean ± SD local intensity of competition as a function of the global intensity of competition (aka population density), for populations of memoryless (dashed line and zebra area) and with-memory (plain line and grey area) individuals. The dark grey dashed line represents the equality relationship for comparative purposes. The variation shown is the mean variation between individuals within simulations.

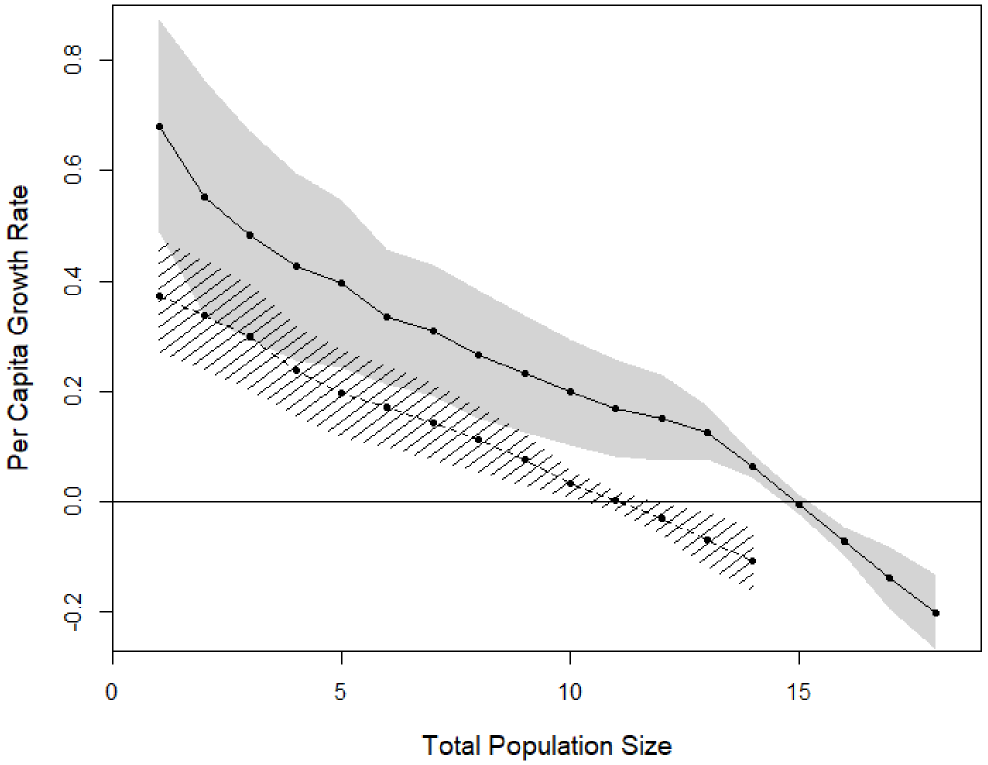

Fig. S5. Mean ± SD (between simulations) per capita growth rate as a function of total population size, for populations of memoryless (dashed line and zebra area) and with-memory individuals (plain line and grey area). Populations made of more than 14 memoryless individuals were never observed.

For populations of with-memory individuals, the best-fitting model is the piecewise 2nd-order polynomial regression (all ΔAIC > 500), with an estimated breakpoint occurring at a population size of 13. At this population size, the negative slope of density-dependence is multiplied by more than 10.

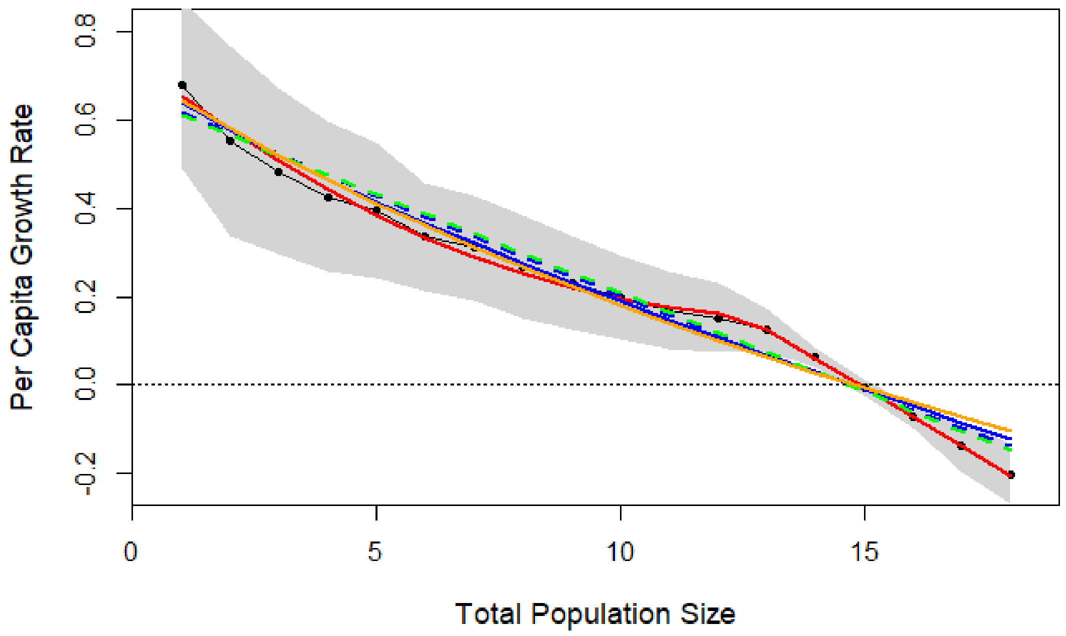

Fig S6. Mean + SD (between simulations) of the per capita growth rate as a function of total population size for populations of with-memory individuals (black line), along with the values predicted by a 2nd-order polynomial regression (dashed blue line), a piecewise 2nd-order polynomial regression (red line), and the Beverton-Holt (orange line), Ricker (dashed green line) and theta-logistic (plain blue line) models.

Supporting Information 2

Sensitivity of the carrying capacity and of the shape of the density-dependence of populations of with-memory individuals to the durations of working (T_W) and reference (T_R) memories.

Carrying capacity

The carrying capacity of populations of with-memory individuals is very robust to changes in the memory parameters T_W and T_R. For T_R ranging between 300 and 1200 and For T_W ranging between 200 and 1000 (with $T_W < T_R$), the carrying capacity varies between 15.1 and 18.2 (Fig. S7).

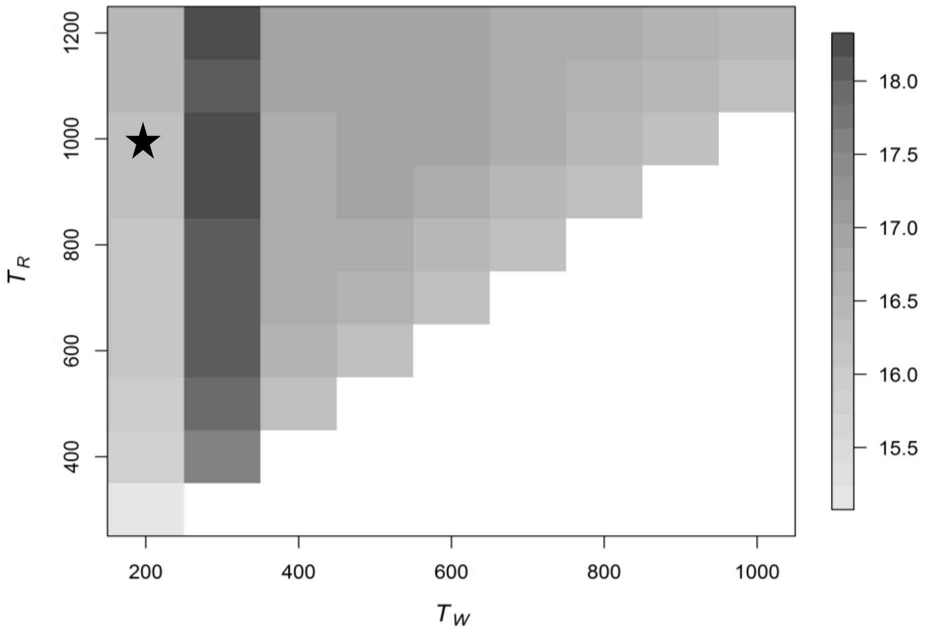

Fig. S7. Mean carrying capacity of populations of with-memory individuals, for different combinations of memory parameters T_W and T_R**,** based on 100 simulations per parameter combination. The black star represents the default parameter configuration.

Shape of the density-dependent response curve

We consider that a breakpoint is present in the density-dependence response curve if the difference in AIC between the with-breakpoint model fitted by nonlinear least squares and the no-breakpoint models is greater than 2. In such cases, we estimate the population size at which the breakpoint occurs and the increase in negative slope that occurs at the breakpoint. When varying the memory parameters T_W between 200 and 1000 and T_R between 300 and 1200 (with $T_W < T_R$), all estimation procedures converge, except for a few combinations of parameters (T_W ; T_R): (300 ; 700), (600 ; 800), (700 ; 1200), (800 ; 1000), and (800 ; 1200), for which no breakpoint is visually present (see Fig. S9). When the model converges, the fit of the segmented polynomial model is better than a simple polynomial, a Beverton-Holt, a Ricker or a theta-logistic model (all ΔAIC > 2) for

all combinations of memory parameters. The location of the breakpoint, when it exists, is very robust and occurs on average for population sizes of 14.5 + 0.8. At the breakpoint, the slope always increases in absolute value; the ratio of slopes after vs before the breakpoint ranging between 1.2 and 14.7, with a mean of 4.8 + 3.5 (Fig S8). The increase of the slope at the breakpoint depends mostly on T_W, with smaller values of T_W leading to a sharper increase in the strength of the negative density-dependence at the breakpoint.

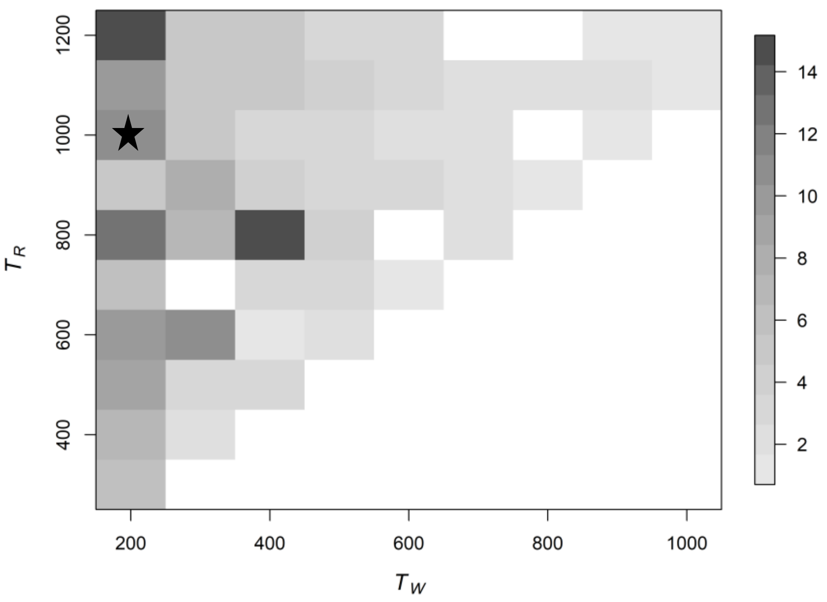

Fig. S8. Ratio of the slopes right after and right before the breakpoint, estimated by nonlinear least squares, for different combinations of memory parameters T_W and T_R based on 100 simulations per parameter combination. White squares in the upper left half of the matrix indicate that the estimation procedure of the breakpoint model by nonlinear least squares did not converge. The black star represents the default parameter configuration.

All density-dependent response curves figures

All panels of Fig. S9 represent the per capita growth rate as a function of the total population size for populations of with-memory individuals (plain line and grey area). We present the curve corresponding to populations of memoryless individuals only for comparative purpose and is the same in all figures, as we allowed memory parameters to vary only.

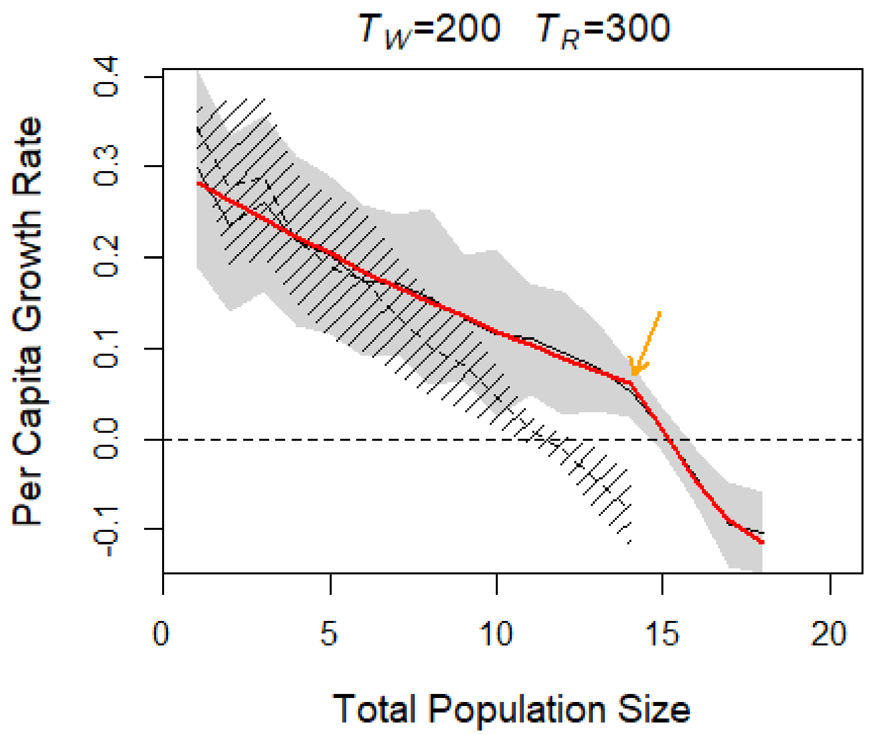
T_W=200 T_R=300
Per Capita Growth Rate
0.4
0.3
0.2
0.1
0.0
-0.1
0
5
10
15
20
Total Population Size

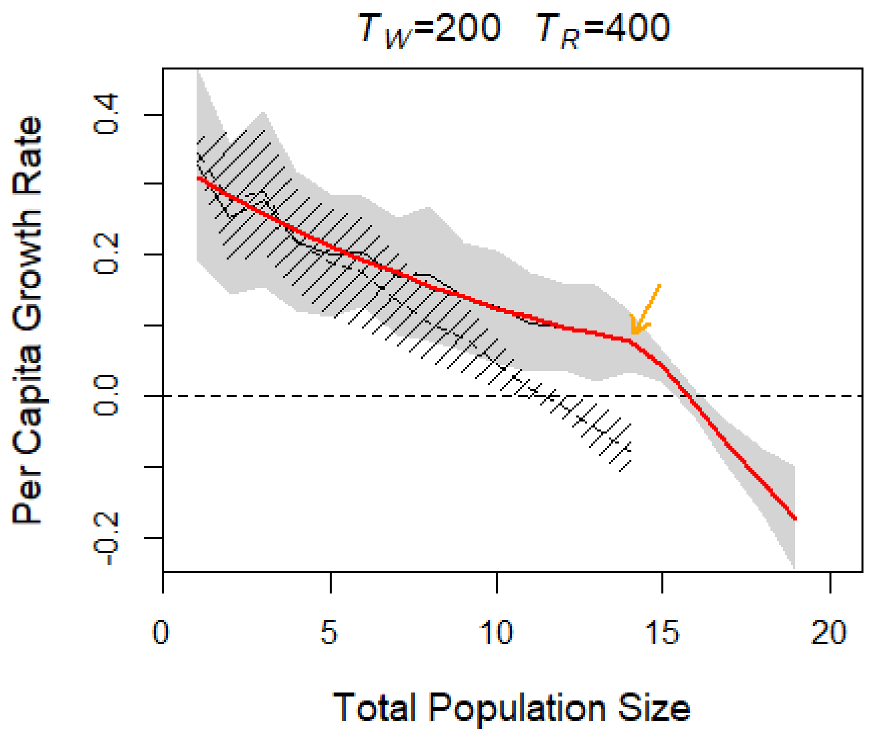
T_W=200 T_R=400
Per Capita Growth Rate
0.4
0.2
0.0
-0.2
0
5
10
15
20
Total Population Size

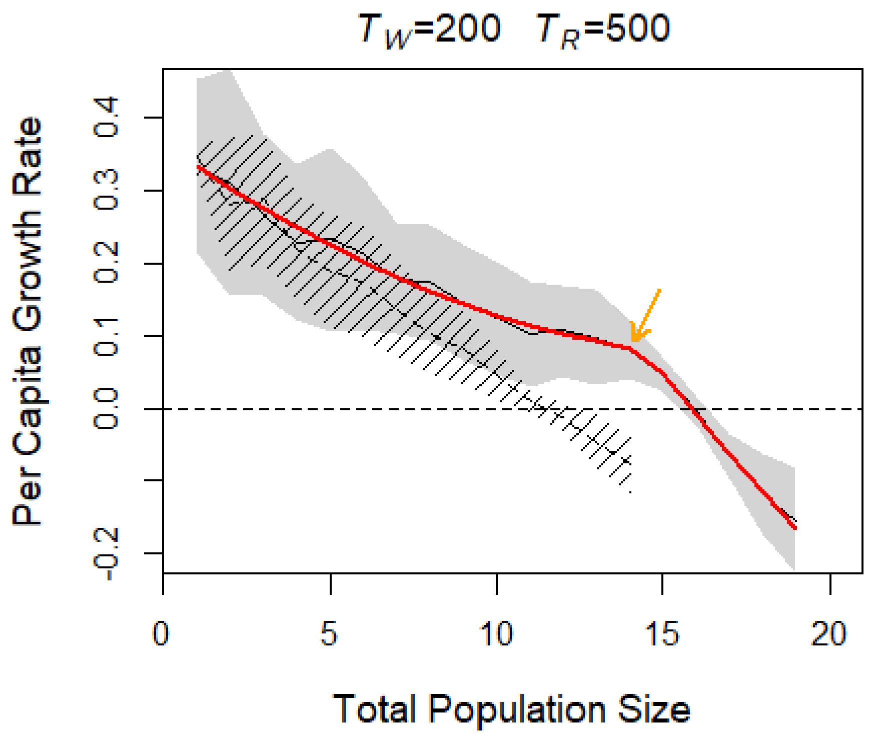
T_W=200 T_R=500
Per Capita Growth Rate
-0.2
0.0
0.1
0.2
0.3
0.4
0
5
10
15
20
Total Population Size

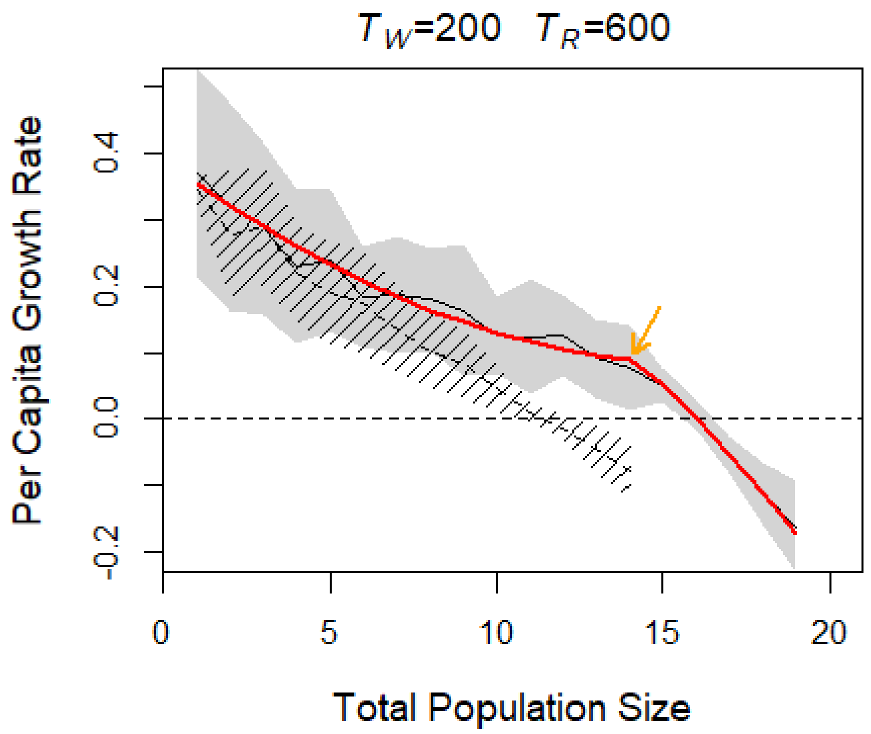
T_W=200 T_R=600
Per Capita Growth Rate
-0.2
0.0
0.2
0.4
0
5
10
15
20
Total Population Size

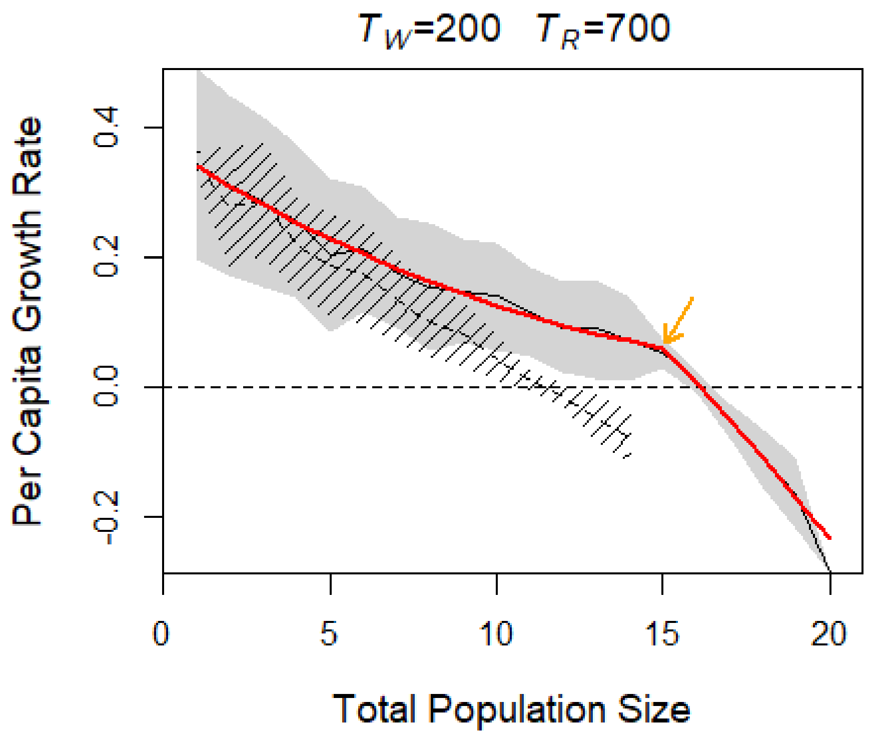
T_W=200 T_R=700
Per Capita Growth Rate
Total Population Size
0.4
0.2
0.0
-0.2
0
5
10
15
20

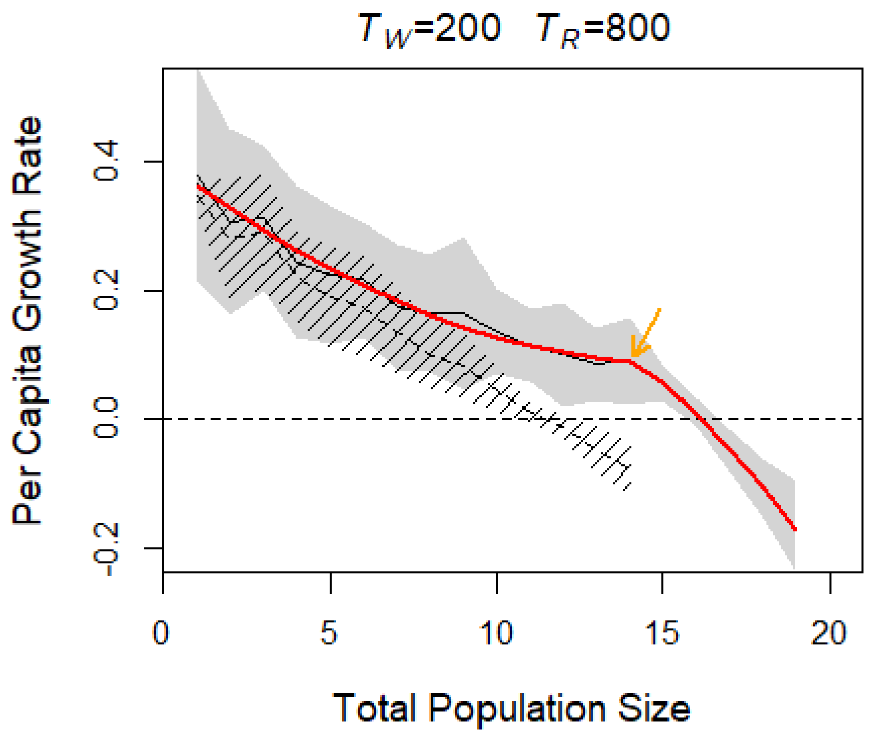
T_W=200 T_R=800
Per Capita Growth Rate
Total Population Size
0.4
0.2
0.0
-0.2
0
5
10
15
20

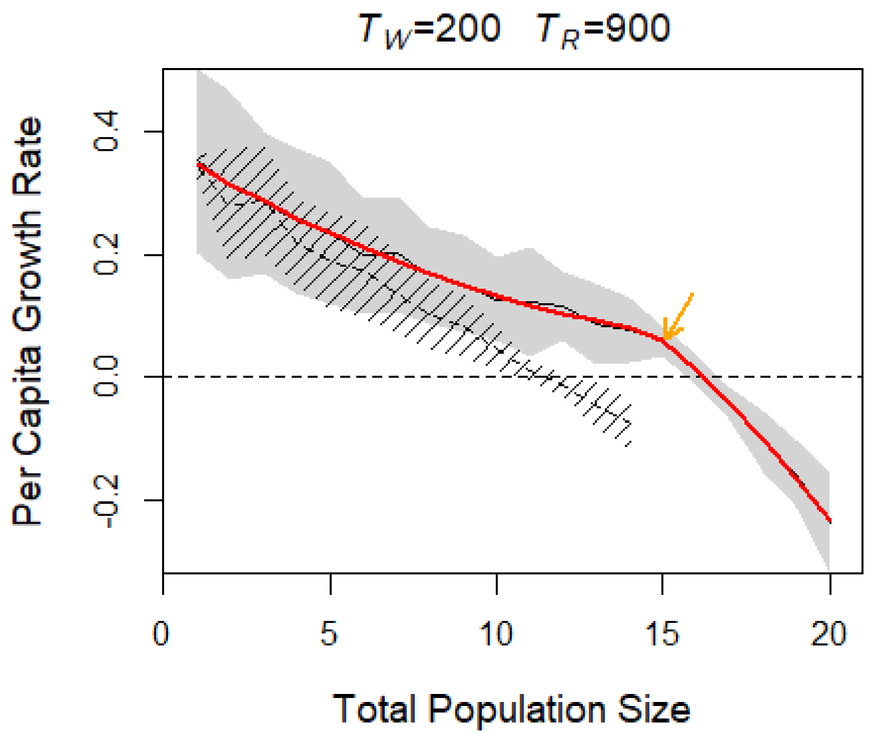
T_W=200 T_R=900
Per Capita Growth Rate
Total Population Size
0.4
0.2
0.0
-0.2
0
5
10
15
20

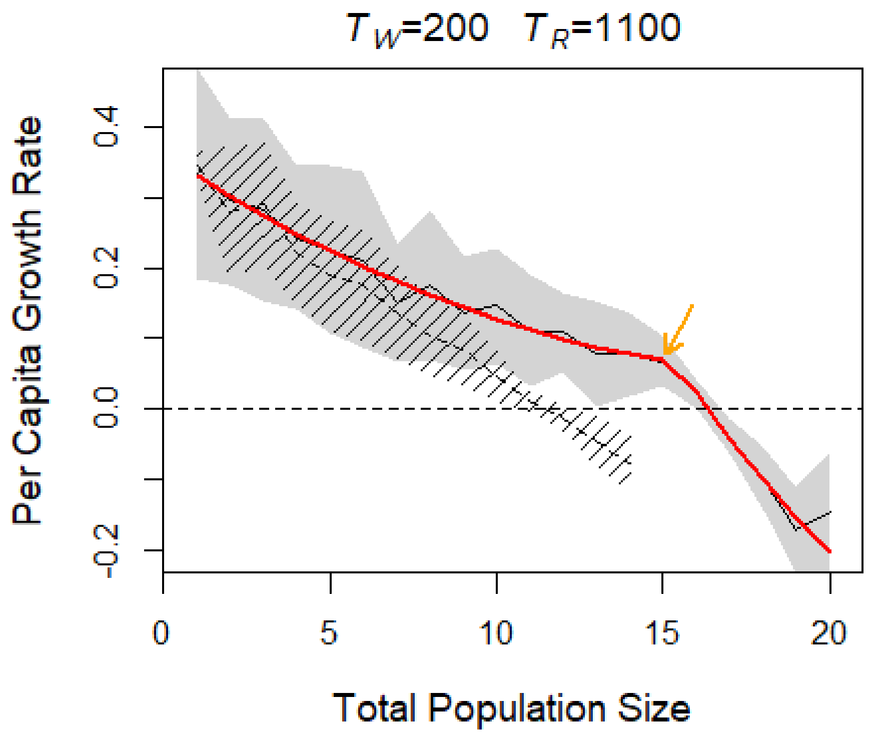
T_W=200 T_R=1100
Per Capita Growth Rate
Total Population Size
0.4
0.2
0.0
-0.2
0
5
10
15
20

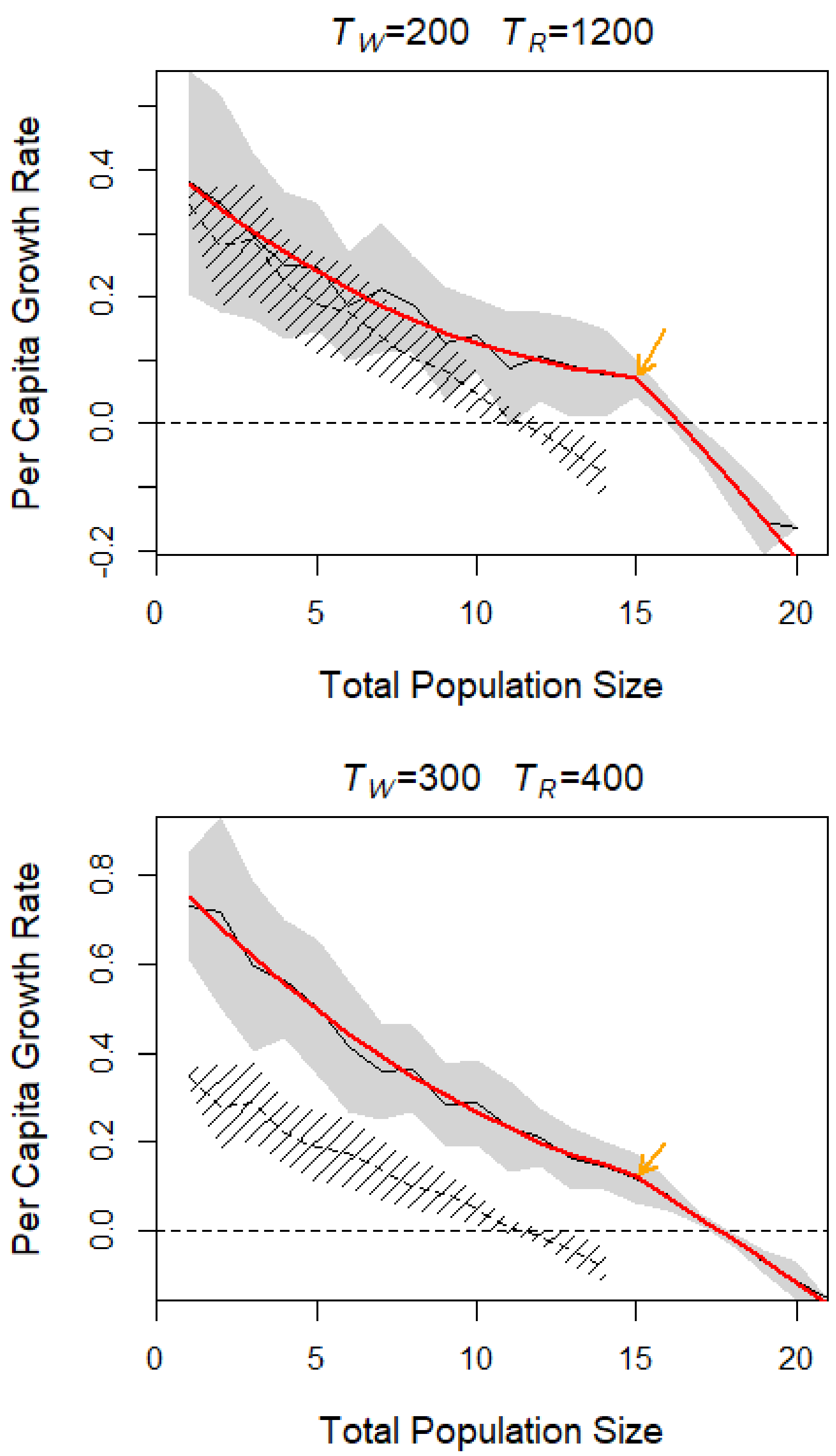

T_W=200 T_R=1200
Per Capita Growth Rate
-0.2
0.0
0.2
0.4
0
5
10
15
20
Total Population Size
T_W=300 T_R=400
Per Capita Growth Rate
0.0
0.2
0.4
0.6
0.8
0
5
10
15
20
Total Population Size

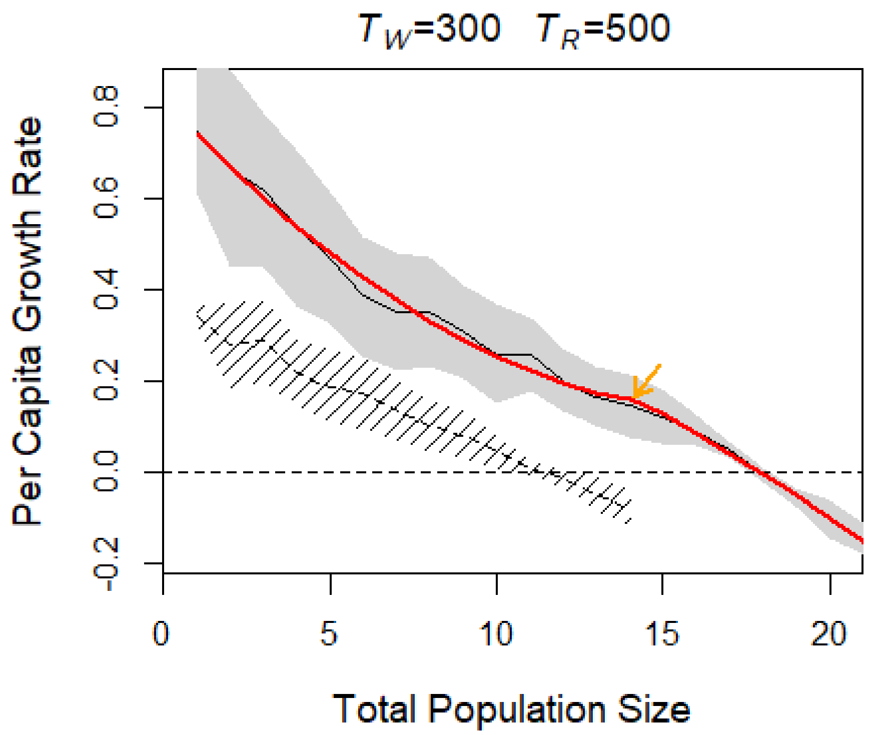

T_W=300 T_R=500
Per Capita Growth Rate
-0.2
0.0
0.2
0.4
0.6
0.8
0
5
10
15
20
Total Population Size

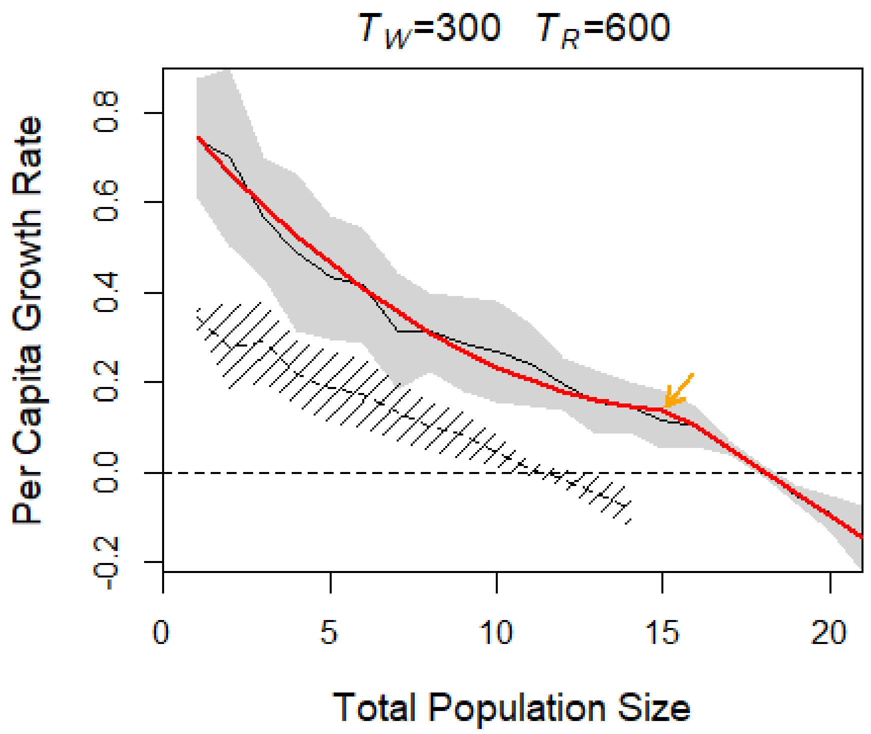

T_W=300 T_R=600
Per Capita Growth Rate
-0.2
0.0
0.2
0.4
0.6
0.8
0
5
10
15
20
Total Population Size

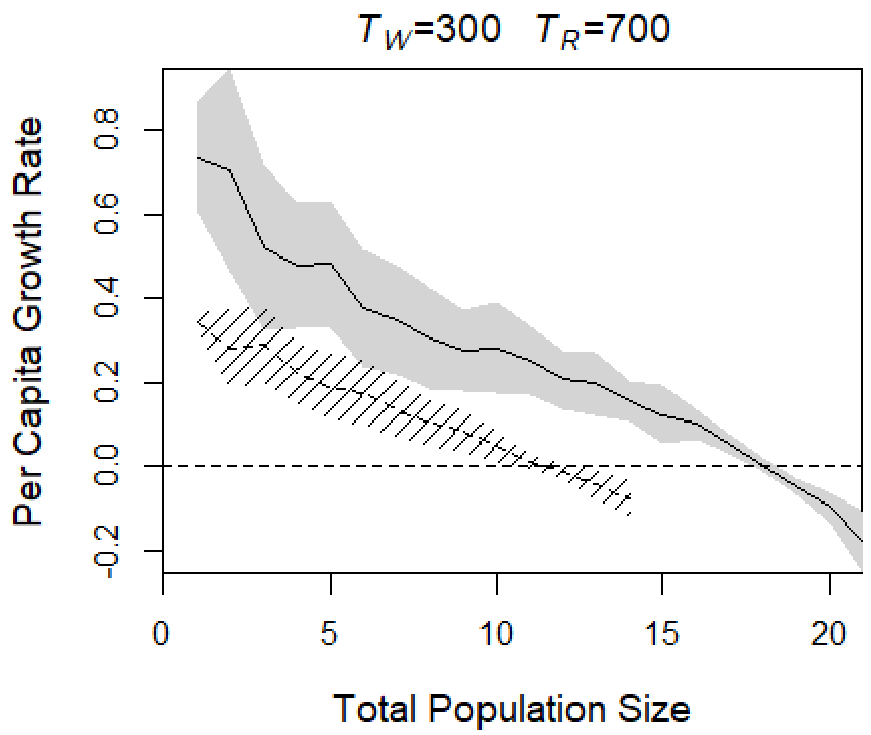
T_W=300 T_R=700
Per Capita Growth Rate
Total Population Size
0
5
10
15
20
-0.2
0.0
0.2
0.4
0.6
0.8

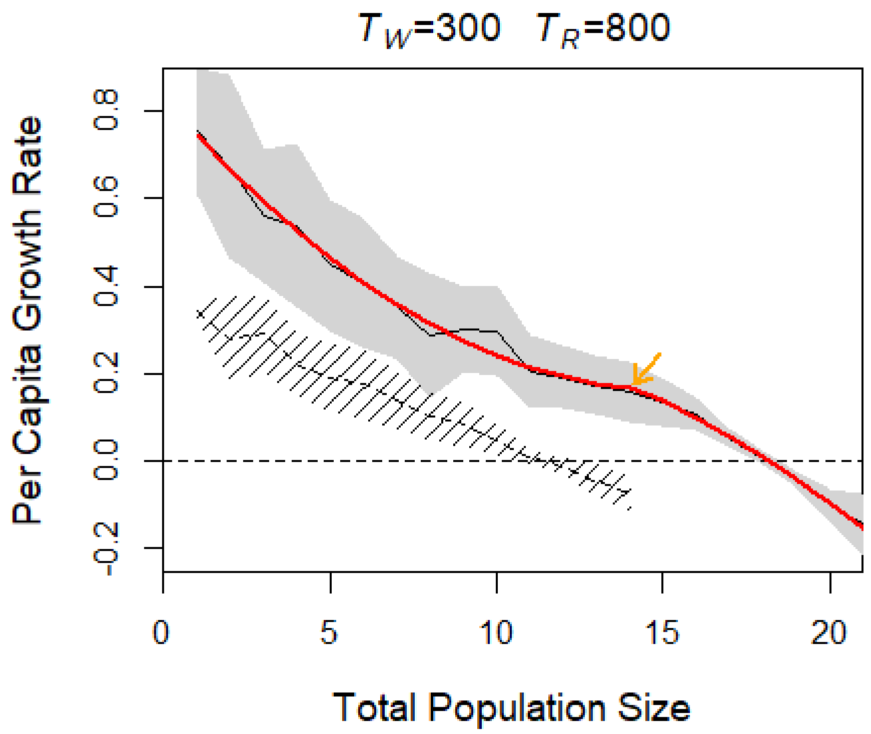
T_W=300 T_R=800
Per Capita Growth Rate
Total Population Size
0
5
10
15
20
-0.2
0.0
0.2
0.4
0.6
0.8

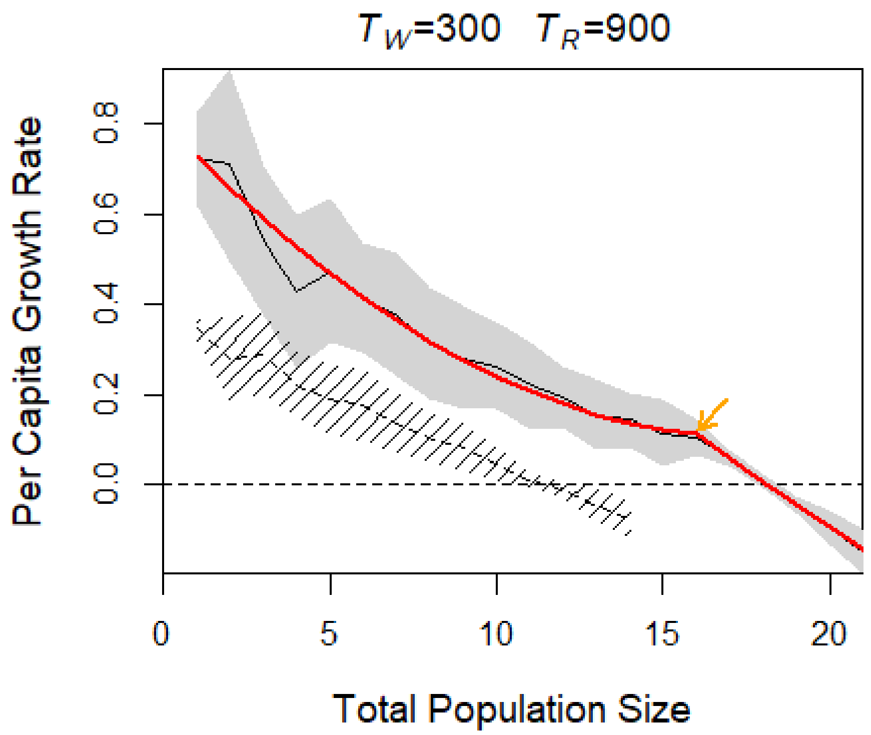
T_W=300 T_R=900
Per Capita Growth Rate
0.0
0.2
0.4
0.6
0.8
0
5
10
15
20
Total Population Size

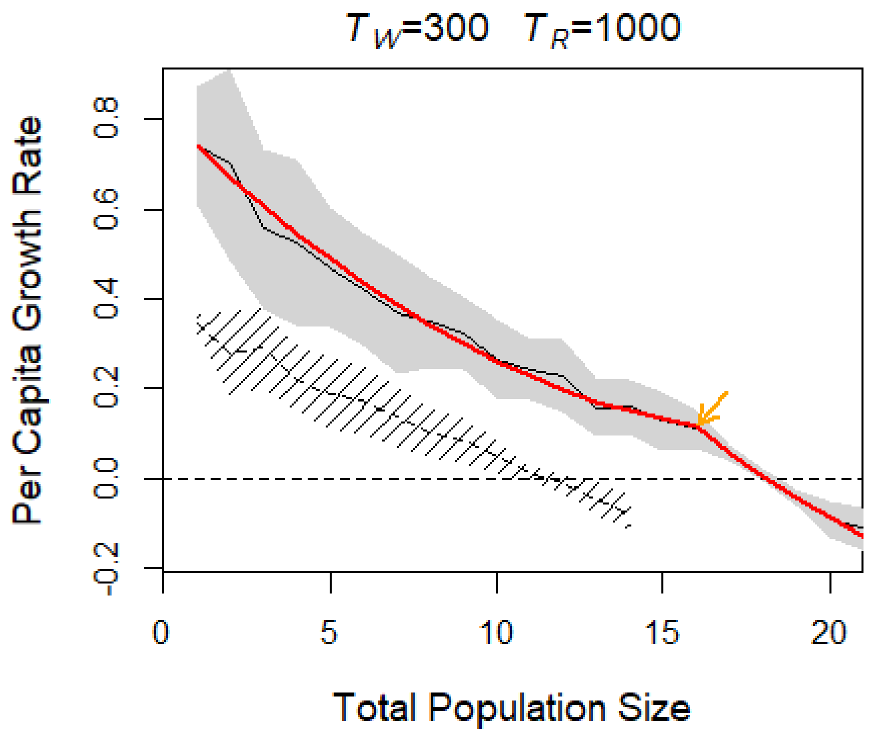
T_W=300 T_R=1000
Per Capita Growth Rate
-0.2
0.0
0.2
0.4
0.6
0.8
0
5
10
15
20
Total Population Size

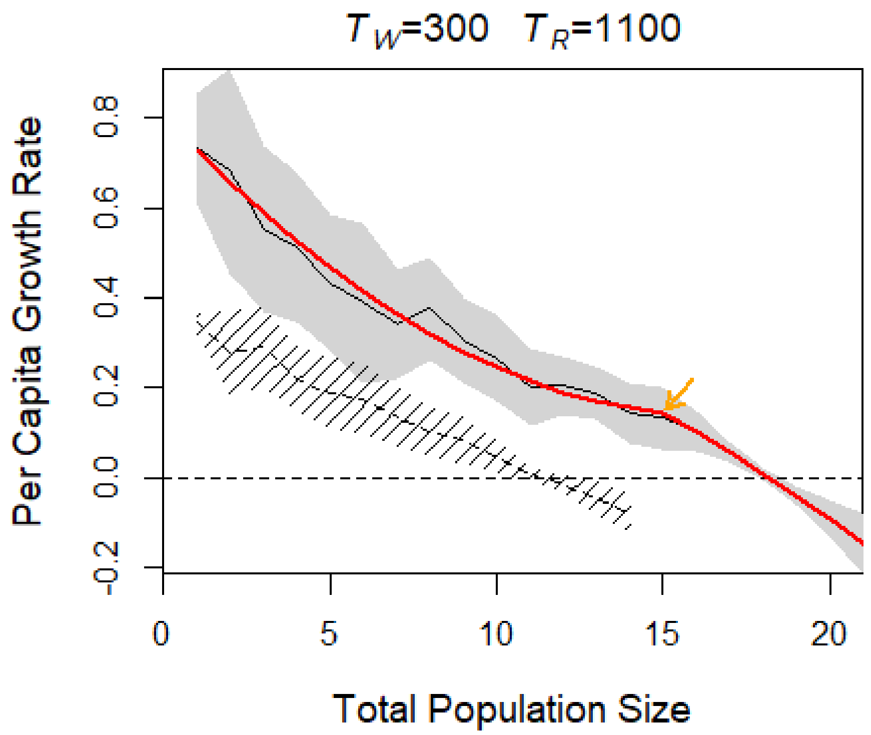
T_W=300 T_R=1100
Per Capita Growth Rate
-0.2
0.0
0.2
0.4
0.6
0.8
0
5
10
15
20
Total Population Size

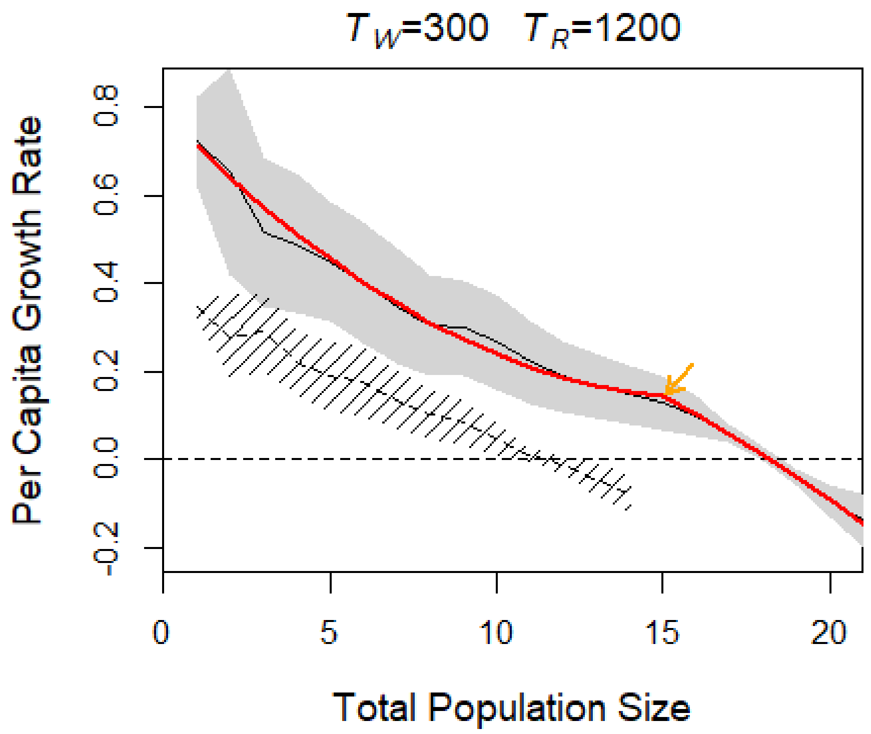
T_W=300 T_R=1200
Per Capita Growth Rate
-0.2
0.0
0.2
0.4
0.6
0.8
0
5
10
15
20
Total Population Size

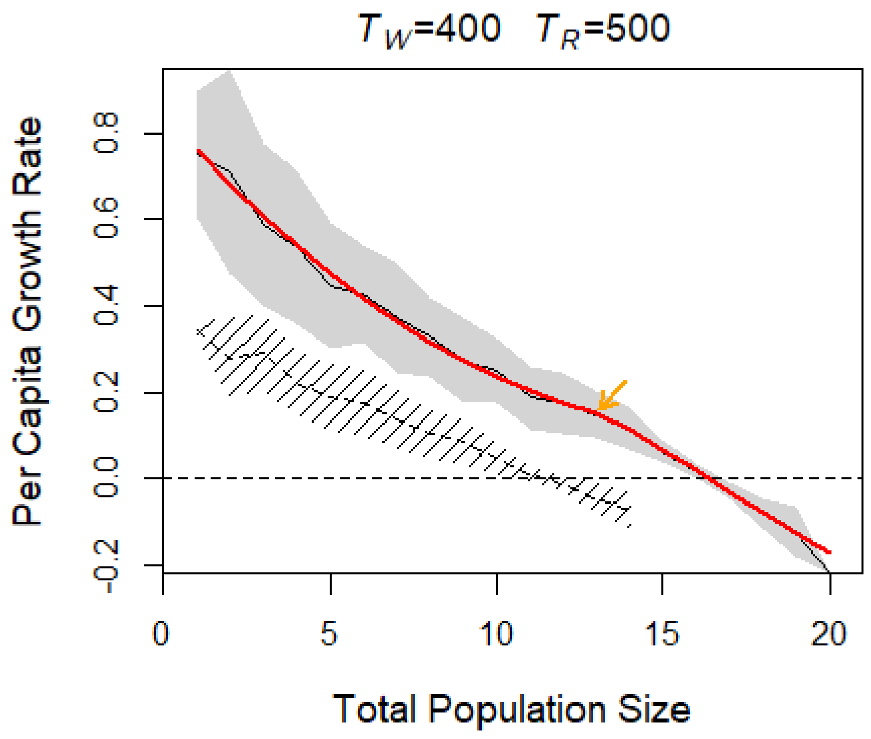

T_W=400 T_R=500
Per Capita Growth Rate
-0.2
0.0
0.2
0.4
0.6
0.8
0
5
10
15
20
Total Population Size

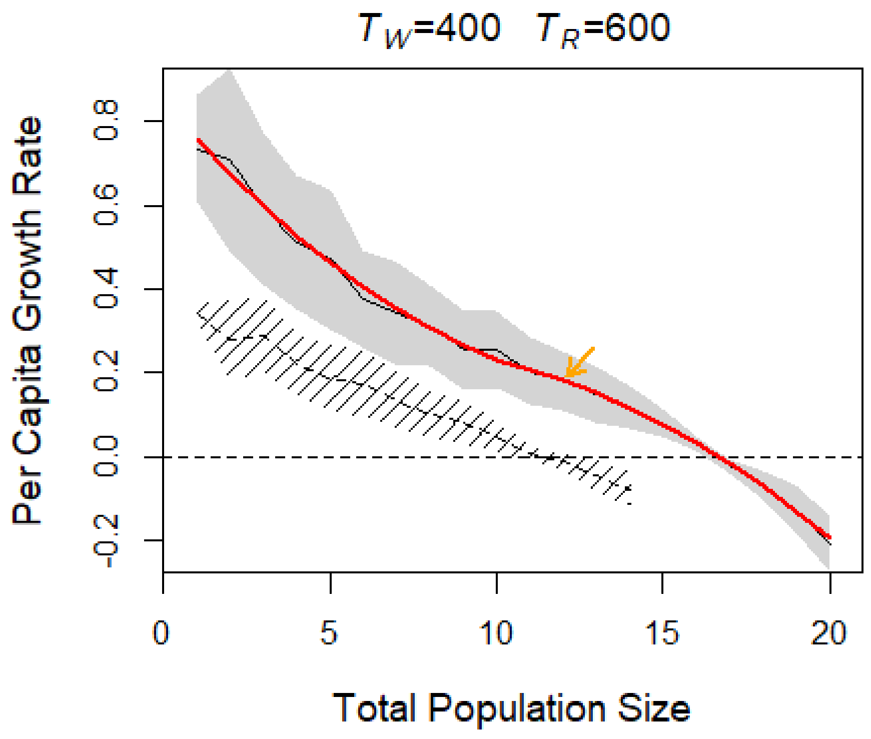

T_W=400 T_R=600
Per Capita Growth Rate
-0.2
0.0
0.2
0.4
0.6
0.8
0
5
10
15
20
Total Population Size

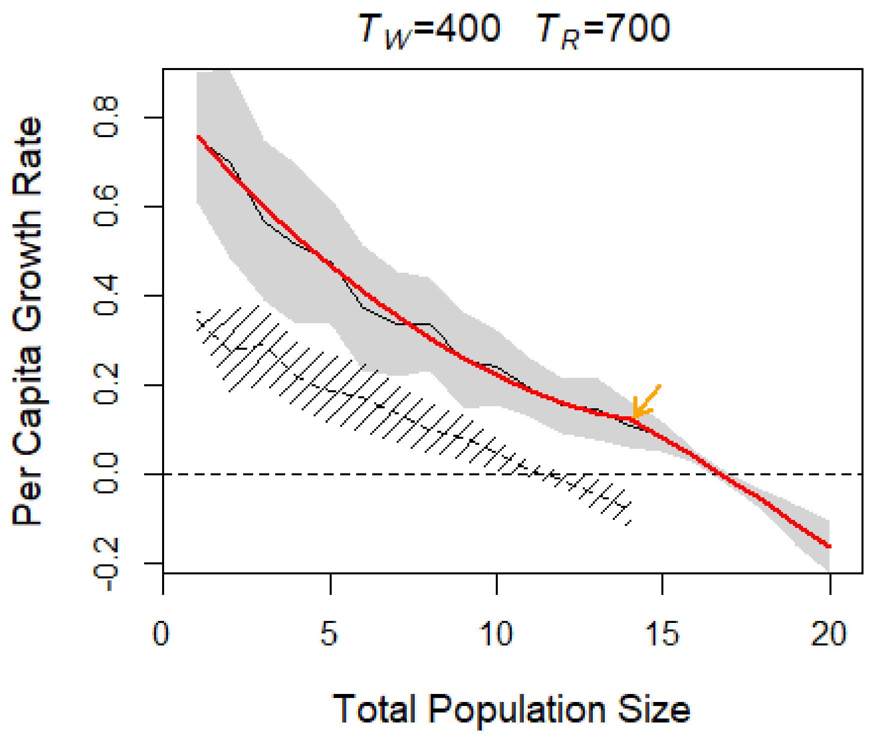
T_W=400 T_R=700
Per Capita Growth Rate
-0.2
0.0
0.2
0.4
0.6
0.8
0
5
10
15
20
Total Population Size

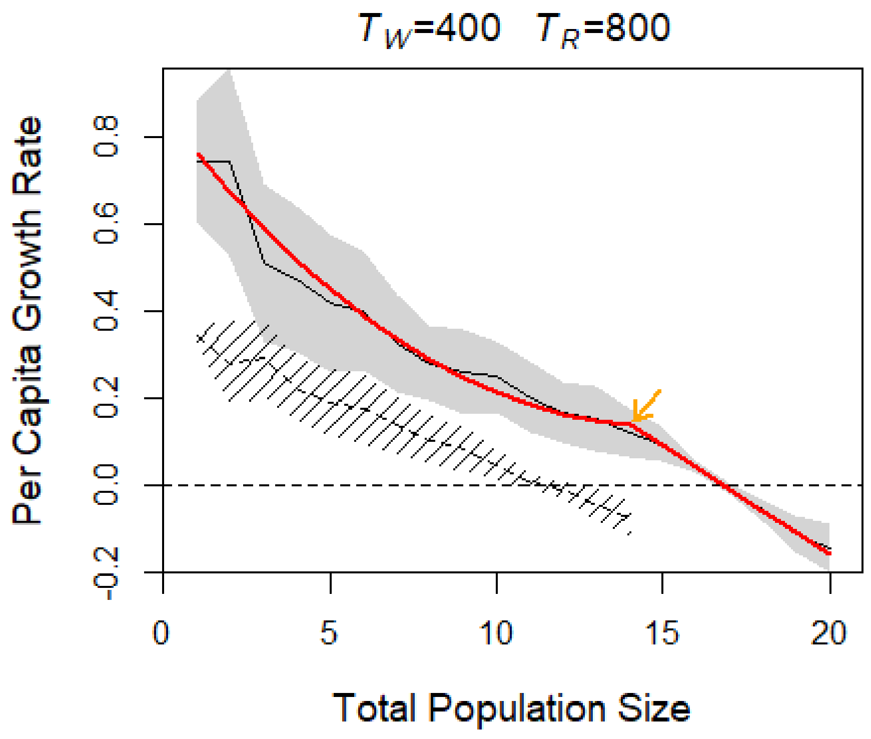
T_W=400 T_R=800
Per Capita Growth Rate
-0.2
0.0
0.2
0.4
0.6
0.8
0
5
10
15
20
Total Population Size

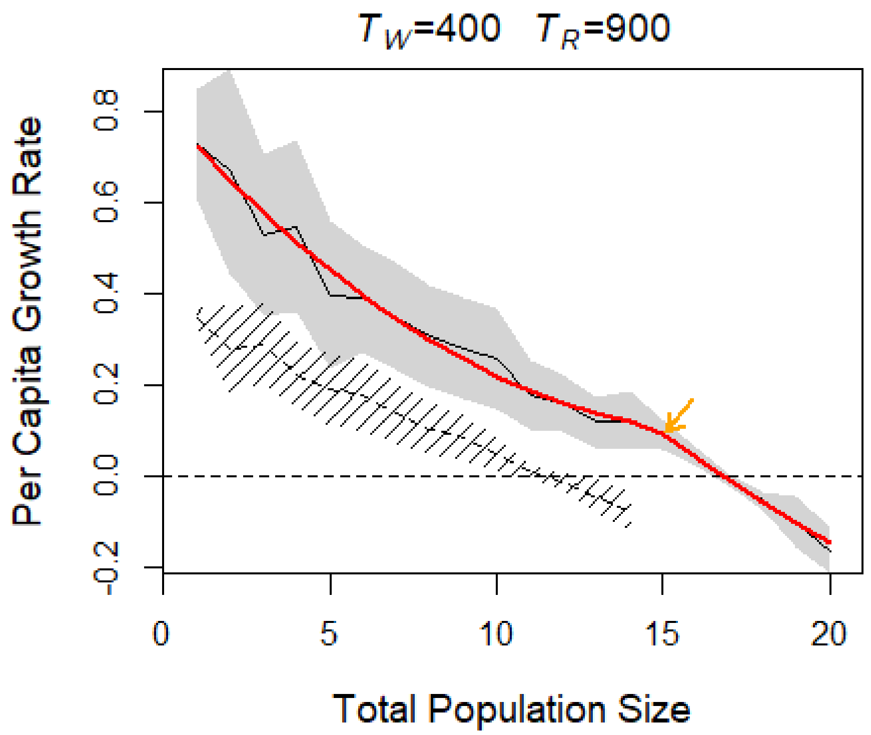
T_W=400 T_R=900
Per Capita Growth Rate
-0.2
0.0
0.2
0.4
0.6
0.8
0
5
10
15
20
Total Population Size

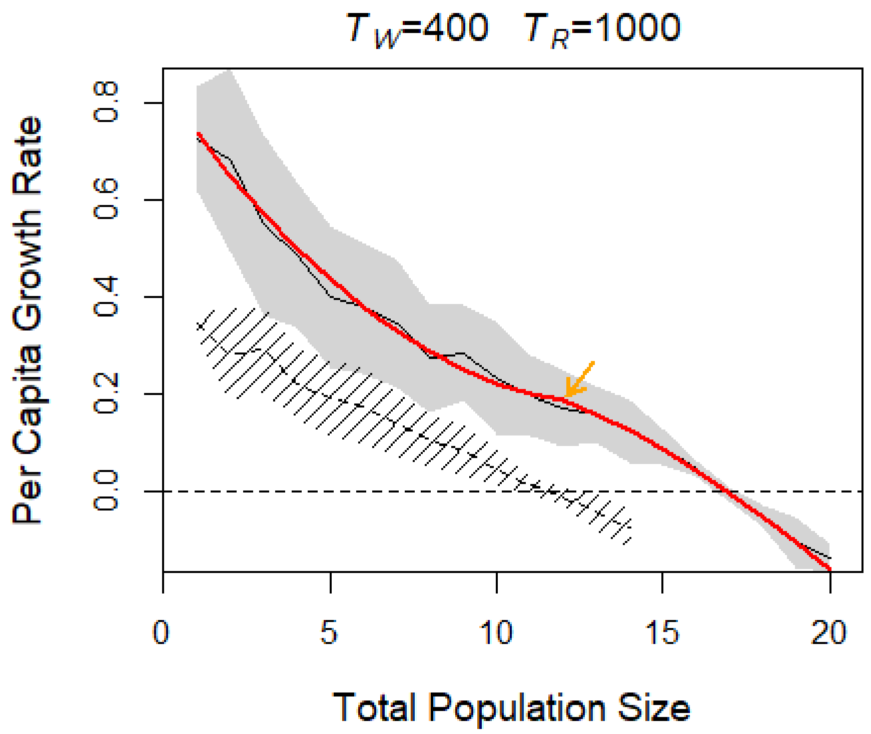
T_W=400 T_R=1000
Per Capita Growth Rate
0.0
0.2
0.4
0.6
0.8
0
5
10
15
20
Total Population Size

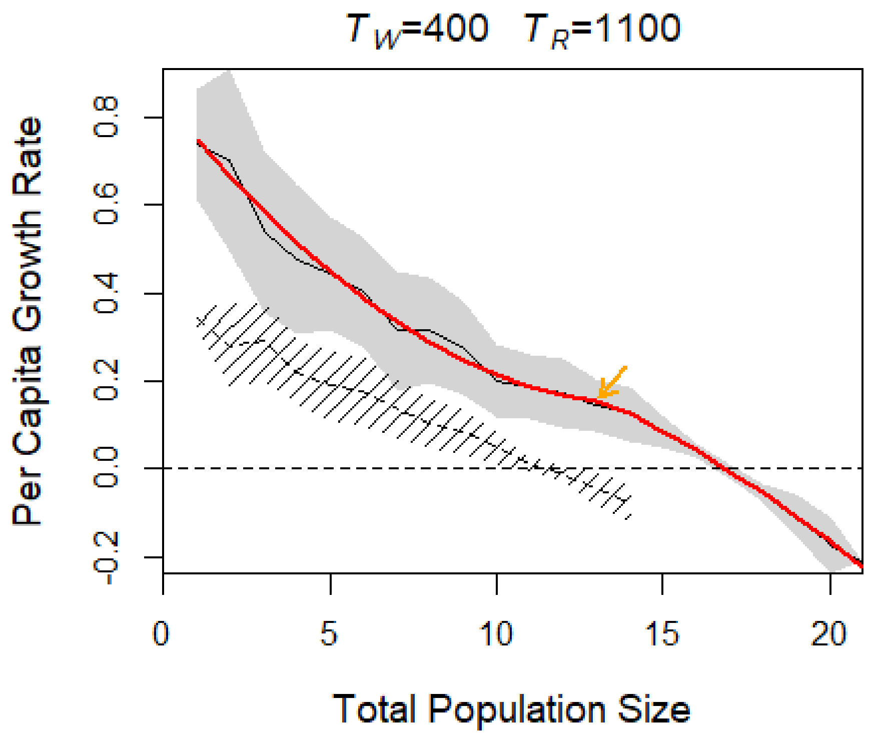
T_W=400 T_R=1100
Per Capita Growth Rate
-0.2
0.0
0.2
0.4
0.6
0.8
0
5
10
15
20
Total Population Size

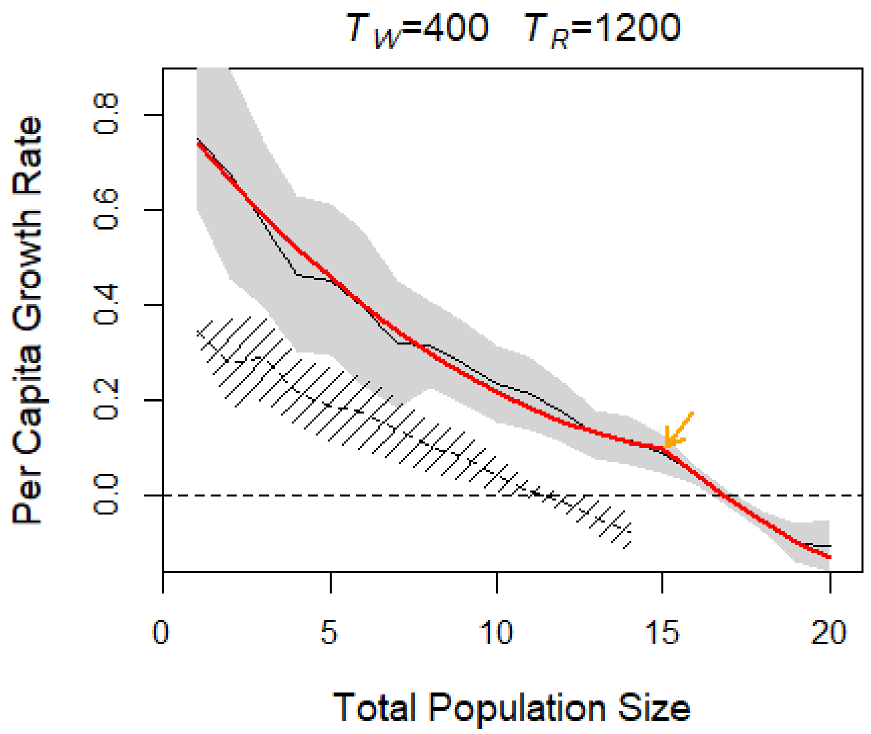
T_W=400 T_R=1200
Per Capita Growth Rate
0.0
0.2
0.4
0.6
0.8
0
5
10
15
20
Total Population Size

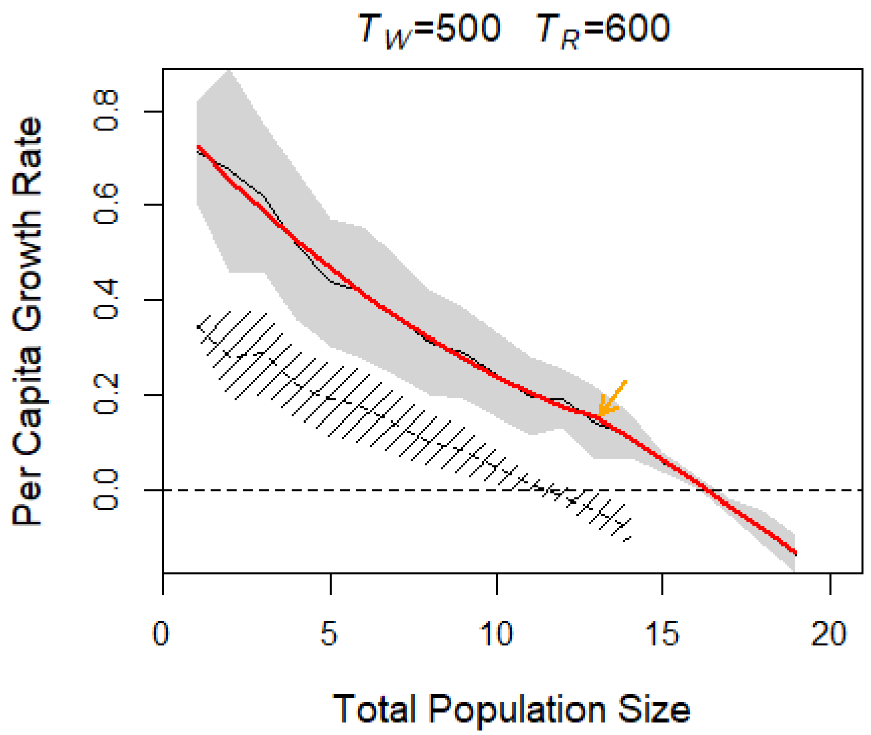
T_W=500 T_R=600
Per Capita Growth Rate
Total Population Size
0.0
0.2
0.4
0.6
0.8
0
5
10
15
20

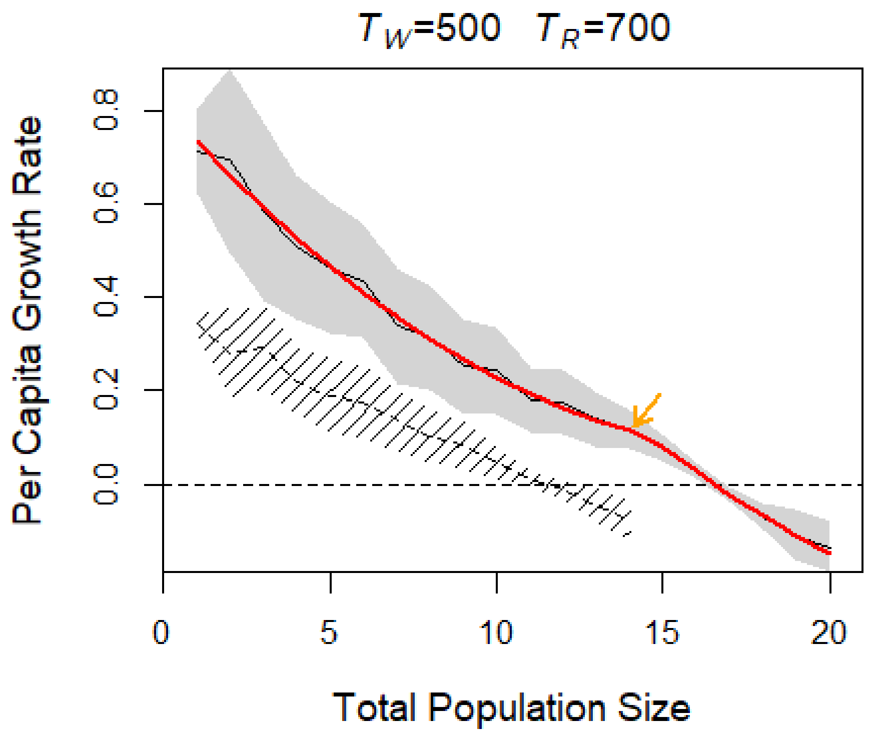
T_W=500 T_R=700
Per Capita Growth Rate
Total Population Size
0.0
0.2
0.4
0.6
0.8
0
5
10
15
20

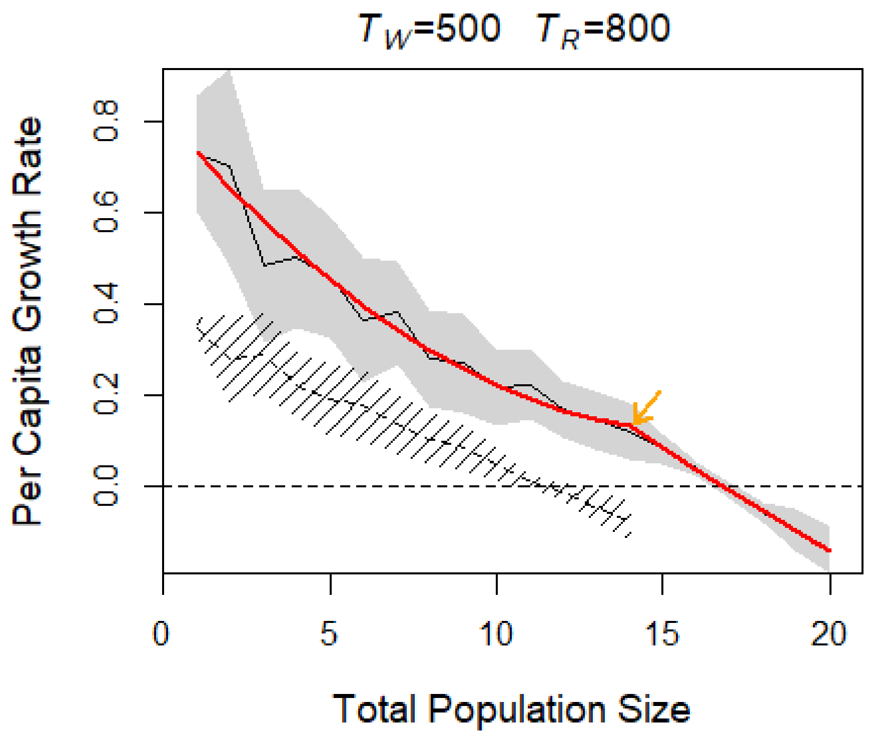

T_W=500 T_R=800
Per Capita Growth Rate
0.0
0.2
0.4
0.6
0.8
0
5
10
15
20
Total Population Size

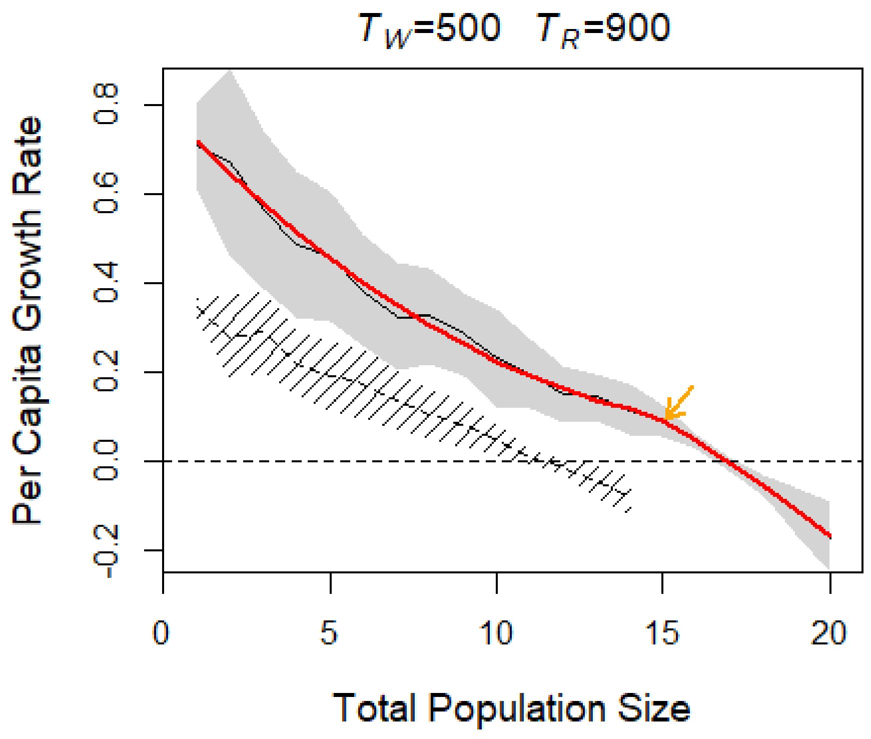

T_W=500 T_R=900
Per Capita Growth Rate
-0.2
0.0
0.2
0.4
0.6
0.8
0
5
10
15
20
Total Population Size

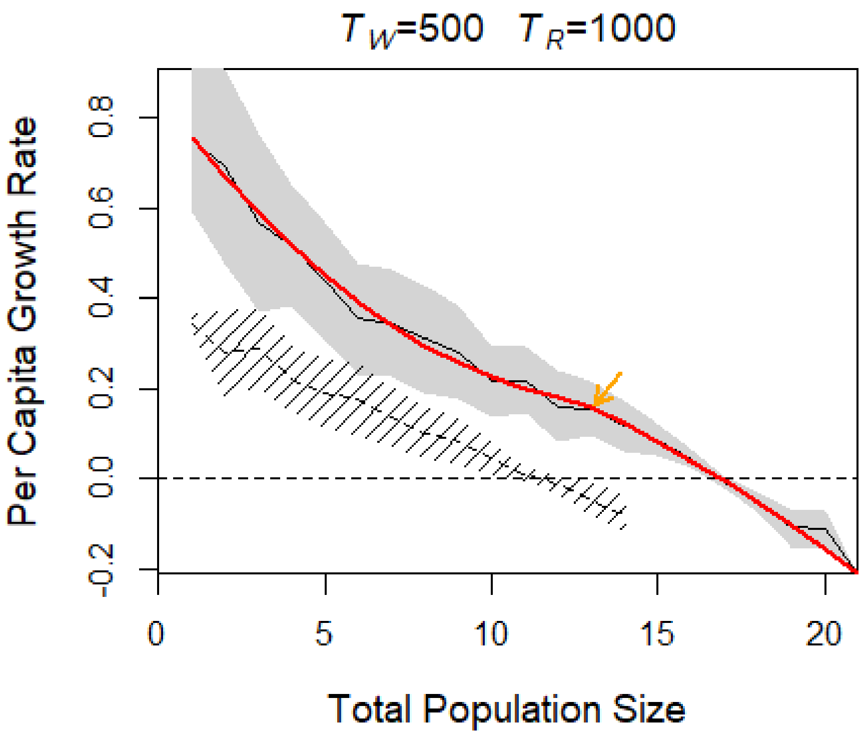
T_W=500 T_R=1000
Per Capita Growth Rate
-0.2
0.0
0.2
0.4
0.6
0.8
0
5
10
15
20
Total Population Size

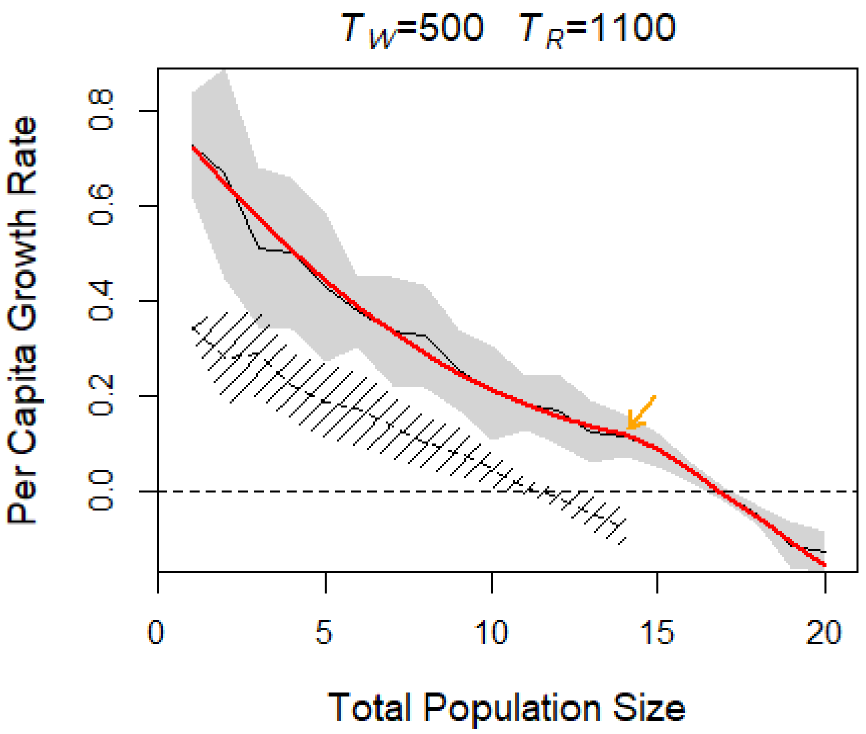
T_W=500 T_R=1100
Per Capita Growth Rate
0.0
0.2
0.4
0.6
0.8
0
5
10
15
20
Total Population Size

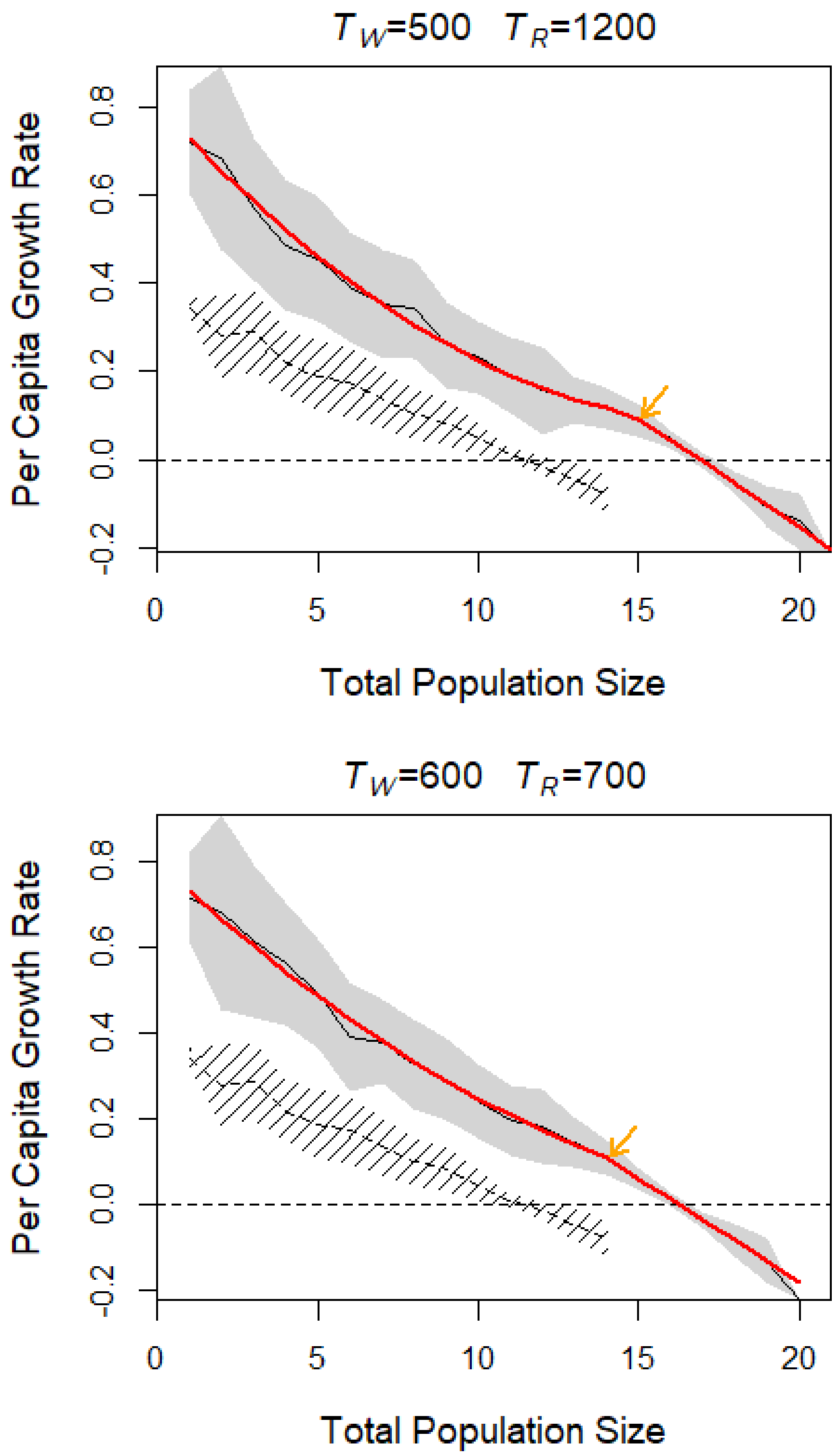
T_W=500 T_R=1200
Per Capita Growth Rate
Total Population Size
T_W=600 T_R=700
Per Capita Growth Rate
Total Population Size

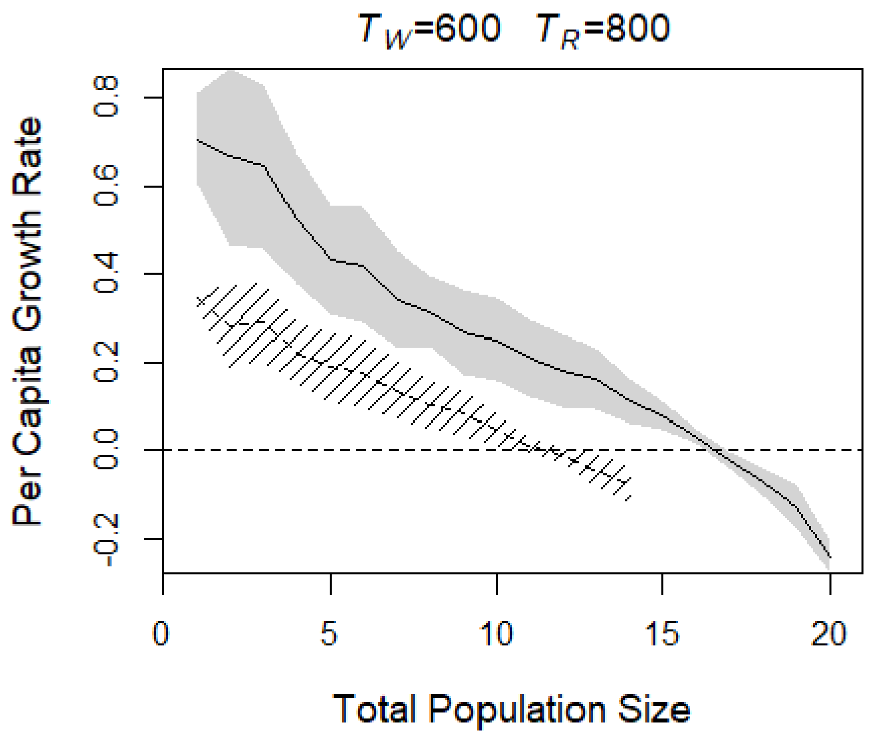
T_W=600 T_R=800
Per Capita Growth Rate
-0.2
0.0
0.2
0.4
0.6
0.8
0
5
10
15
20
Total Population Size

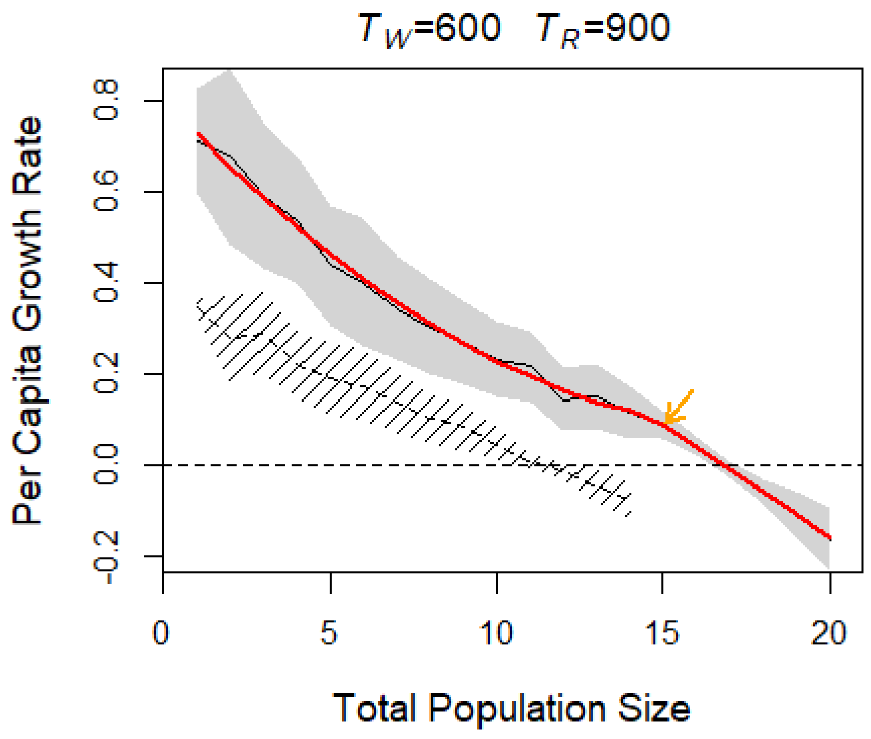
T_W=600 T_R=900
Per Capita Growth Rate
-0.2
0.0
0.2
0.4
0.6
0.8
0
5
10
15
20
Total Population Size

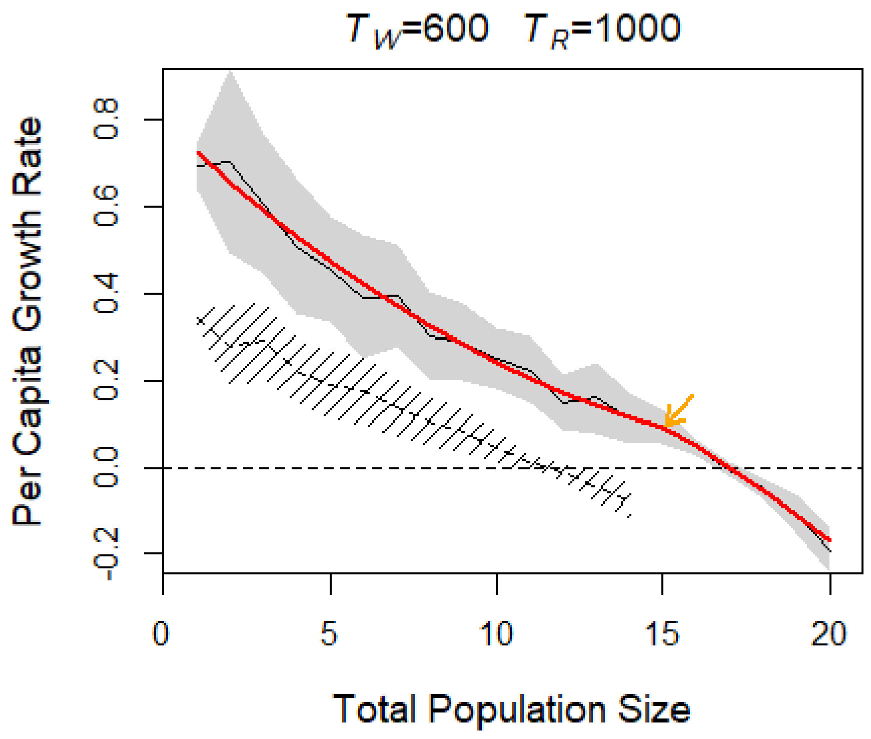

T_W=600 T_R=1000
Per Capita Growth Rate
-0.2
0.0
0.2
0.4
0.6
0.8
0
5
10
15
20
Total Population Size

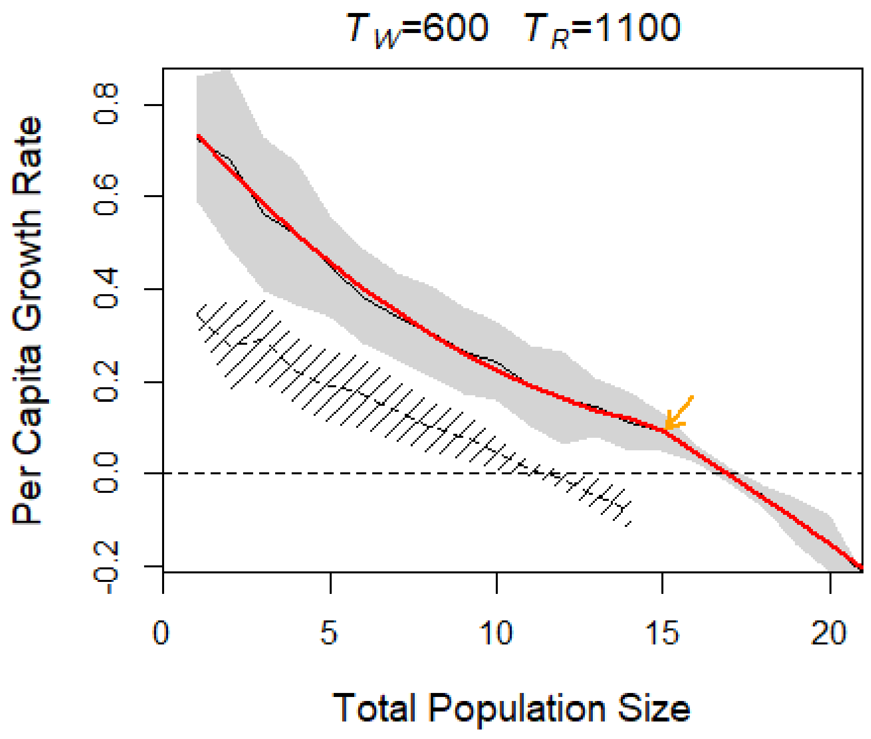

T_W=600 T_R=1100
Per Capita Growth Rate
-0.2
0.0
0.2
0.4
0.6
0.8
0
5
10
15
20
Total Population Size

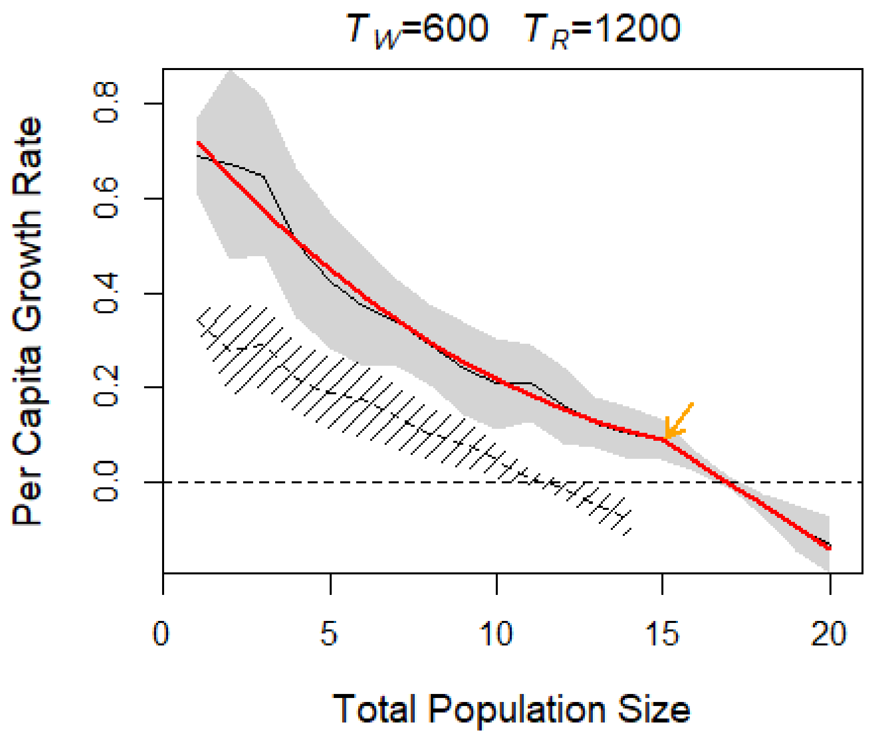

T_W=600 T_R=1200
Per Capita Growth Rate
0.0
0.2
0.4
0.6
0.8
0
5
10
15
20
Total Population Size

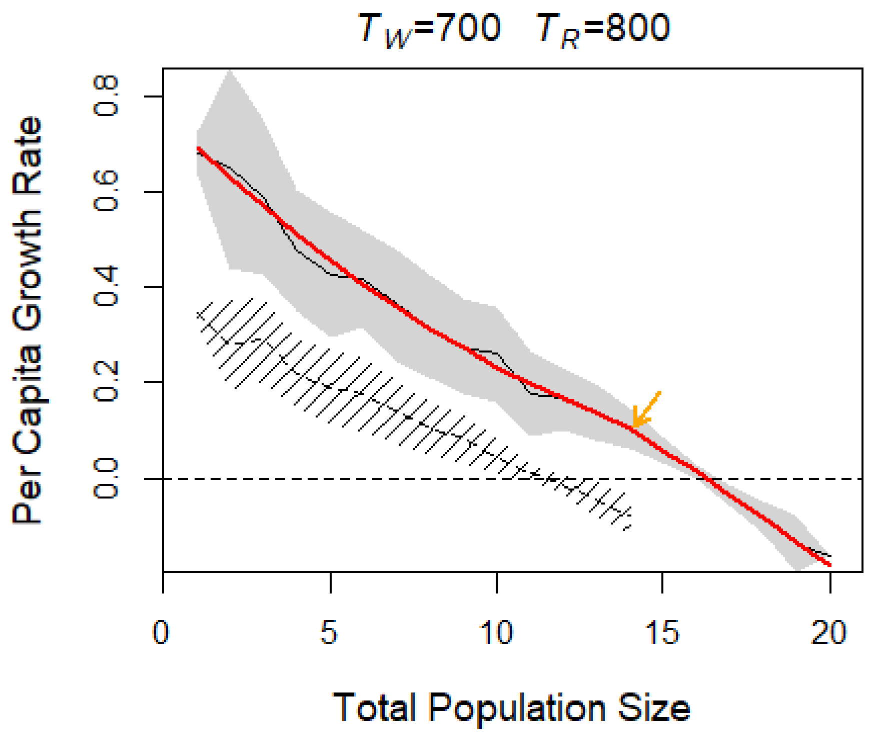

T_W=700 T_R=800
Per Capita Growth Rate
0.0
0.2
0.4
0.6
0.8
0
5
10
15
20
Total Population Size

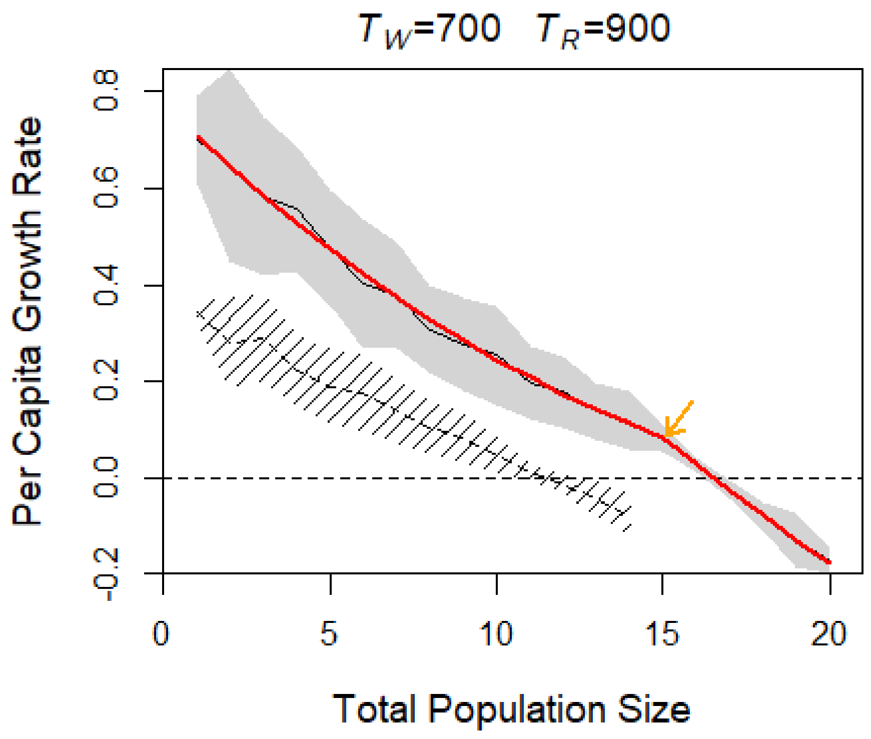

T_W=700 T_R=900
Per Capita Growth Rate
Total Population Size
0.8
0.6
0.4
0.2
0.0
-0.2
0
5
10
15
20

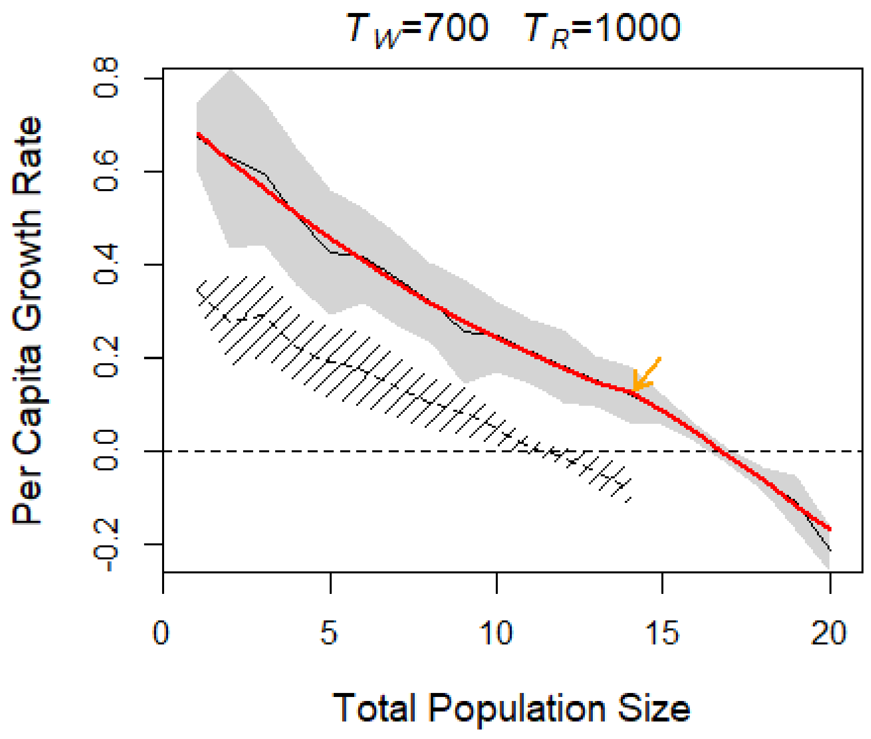

T_W=700 T_R=1000
Per Capita Growth Rate
Total Population Size
0.8
0.6
0.4
0.2
0.0
-0.2
0
5
10
15
20

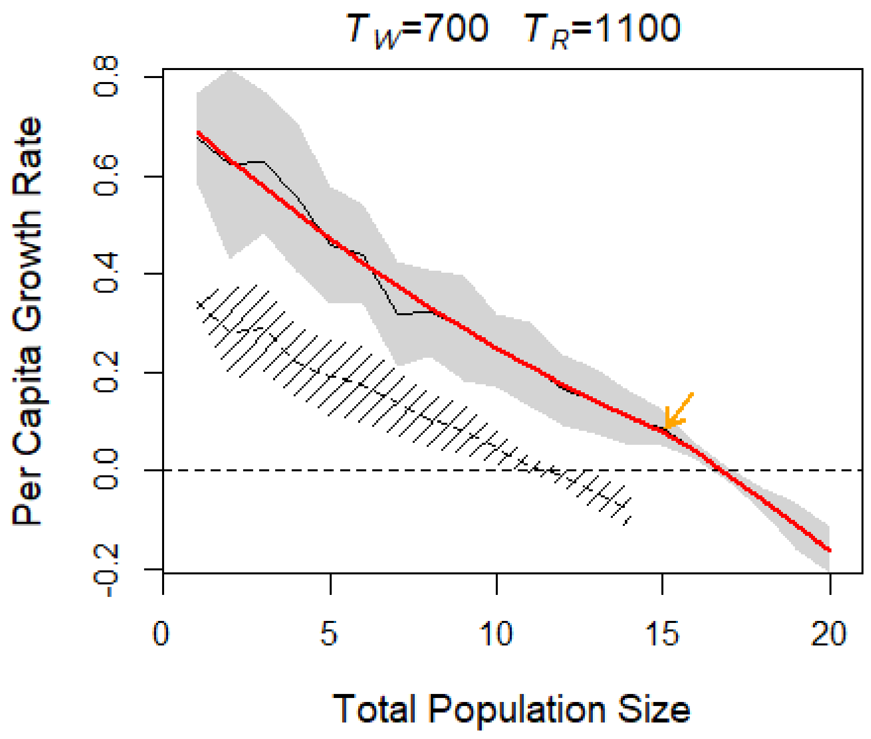
T_W=700 T_R=1100
Per Capita Growth Rate
-0.2
0.0
0.2
0.4
0.6
0.8
0
5
10
15
20
Total Population Size

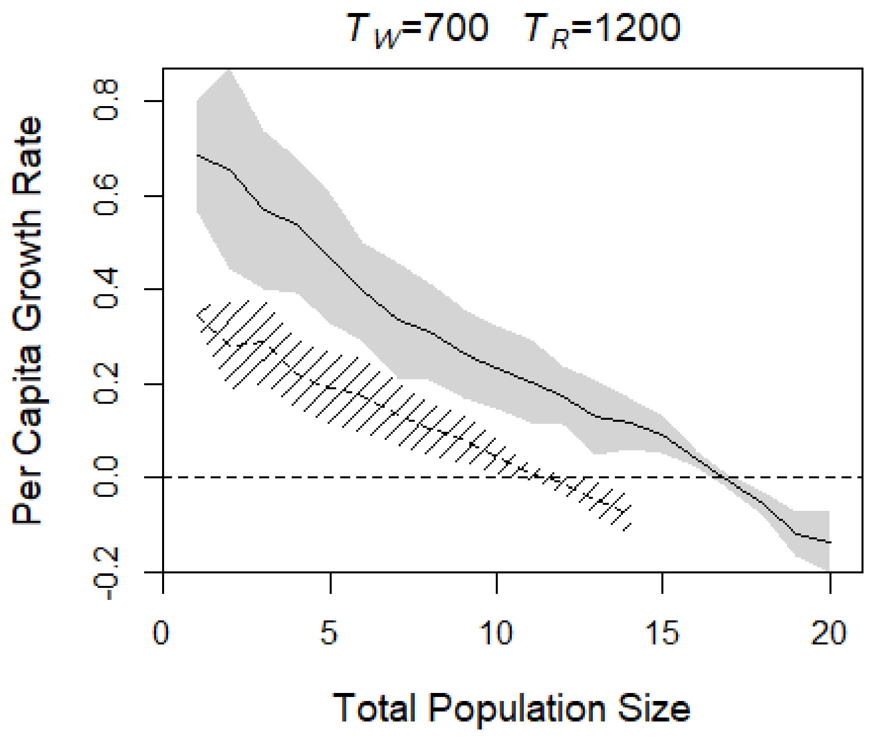
T_W=700 T_R=1200
Per Capita Growth Rate
-0.2
0.0
0.2
0.4
0.6
0.8
0
5
10
15
20
Total Population Size

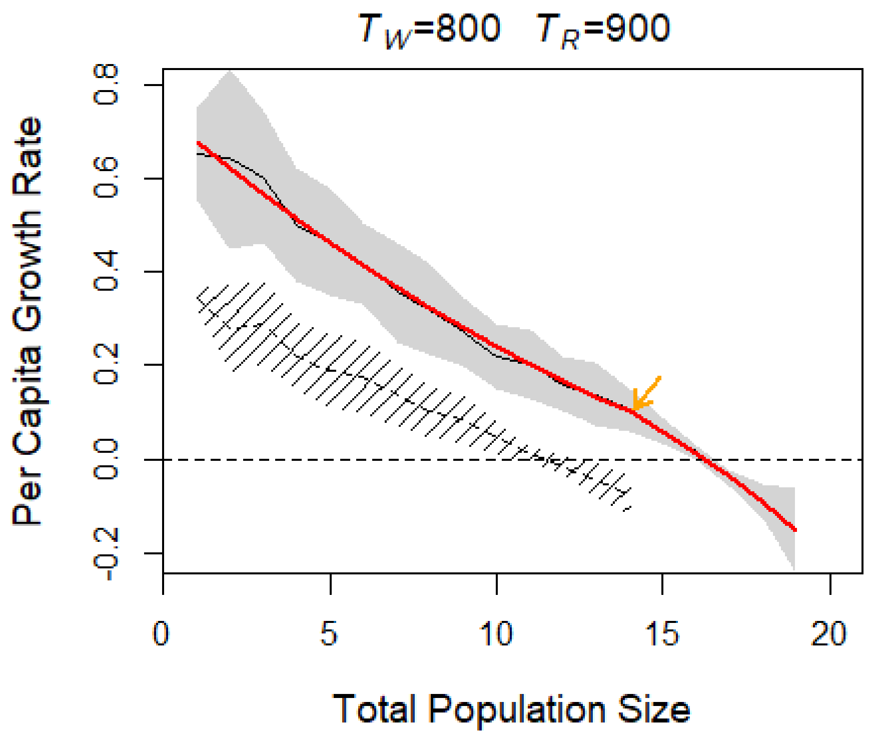
T_W=800 T_R=900
Per Capita Growth Rate
Total Population Size
0.8
0.6
0.4
0.2
0.0
-0.2
0
5
10
15
20

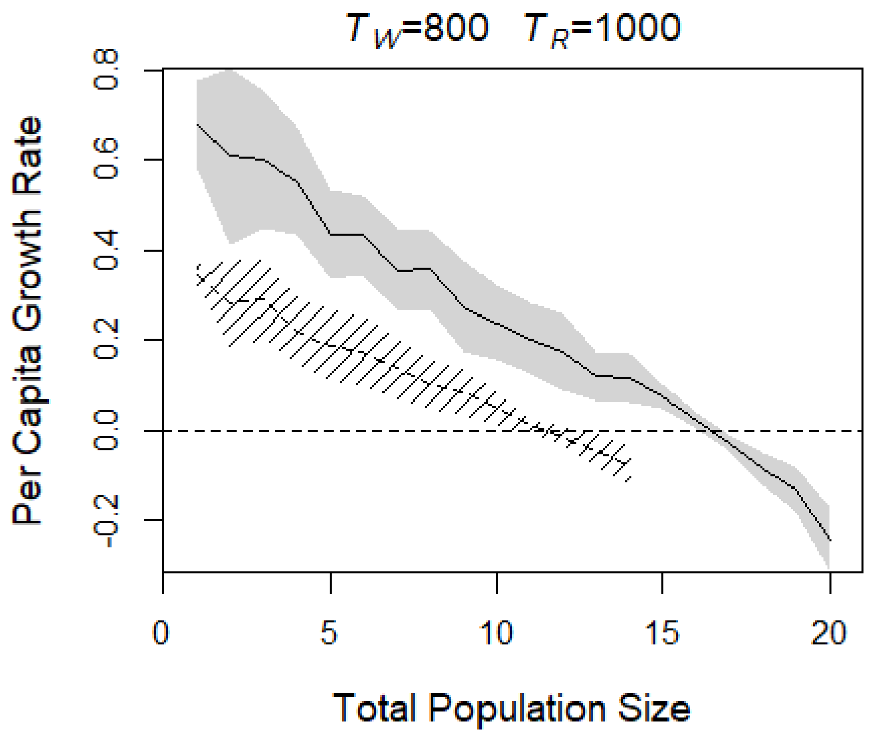
T_W=800 T_R=1000
Per Capita Growth Rate
Total Population Size
0.8
0.6
0.4
0.2
0.0
-0.2
0
5
10
15
20

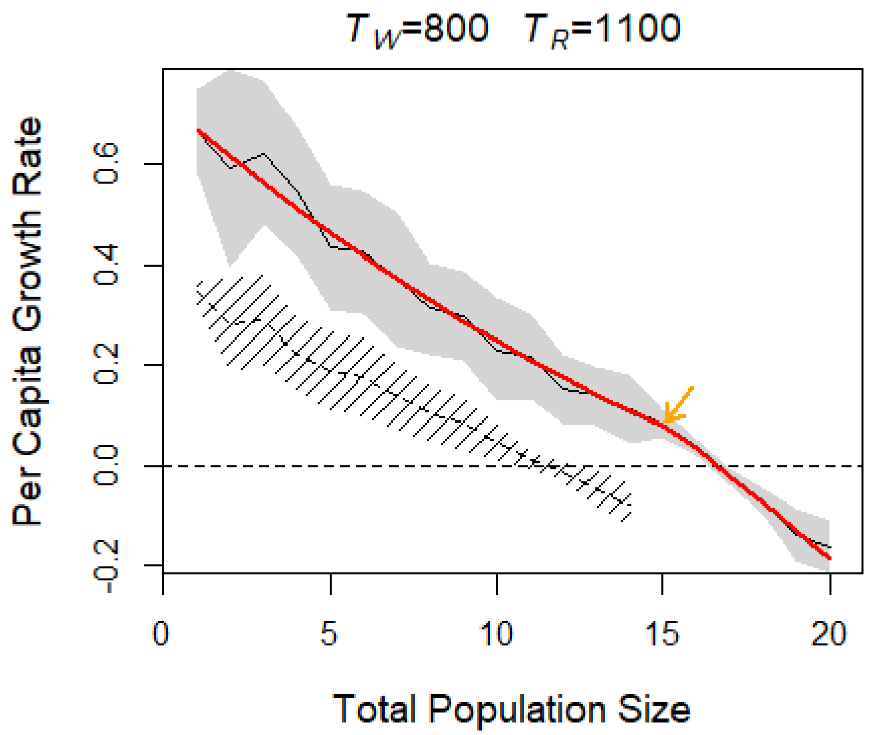

T W=800 T R=1100
Per Capita Growth Rate
-0.2
0.0
0.2
0.4
0.6
0
5
10
15
20
Total Population Size

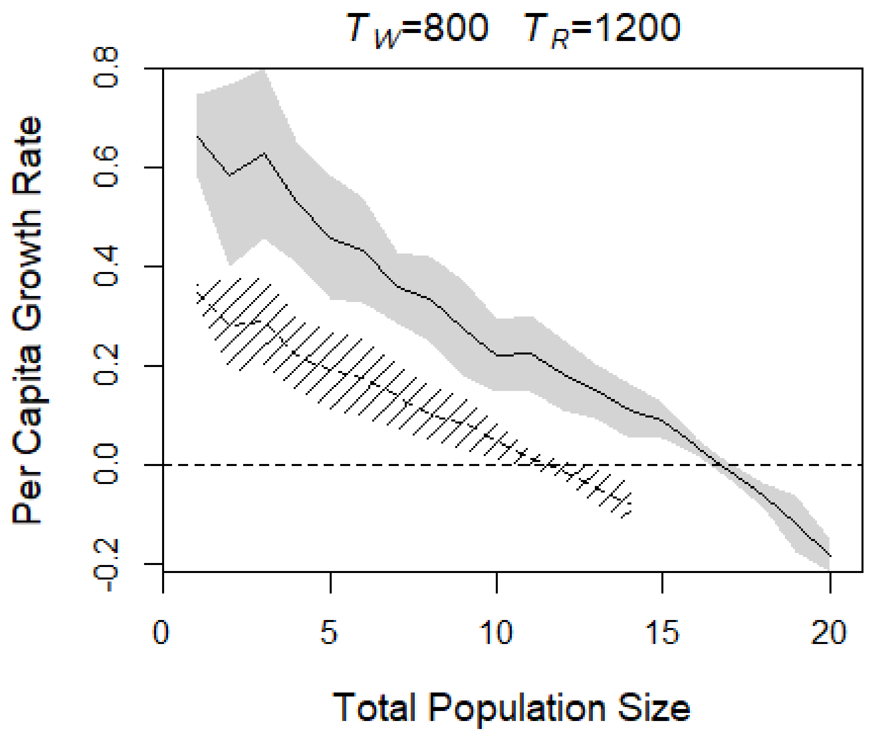

T W=800 T R=1200
Per Capita Growth Rate
-0.2
0.0
0.2
0.4
0.6
0.8
0
5
10
15
20
Total Population Size

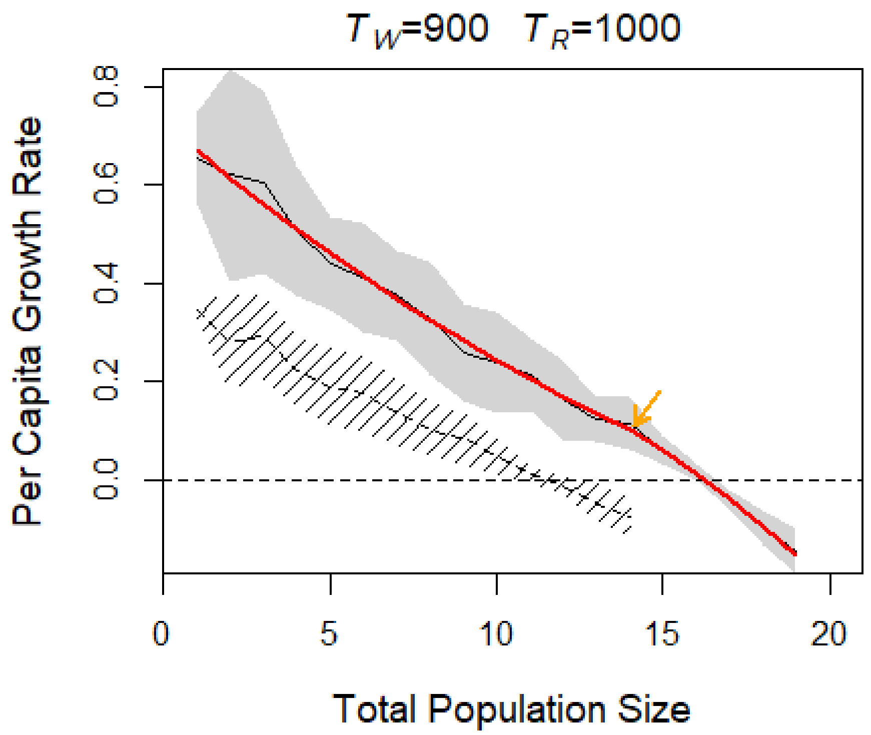

T_W=900 T_R=1000
Per Capita Growth Rate
0.0
0.2
0.4
0.6
0.8
0
5
10
15
20
Total Population Size

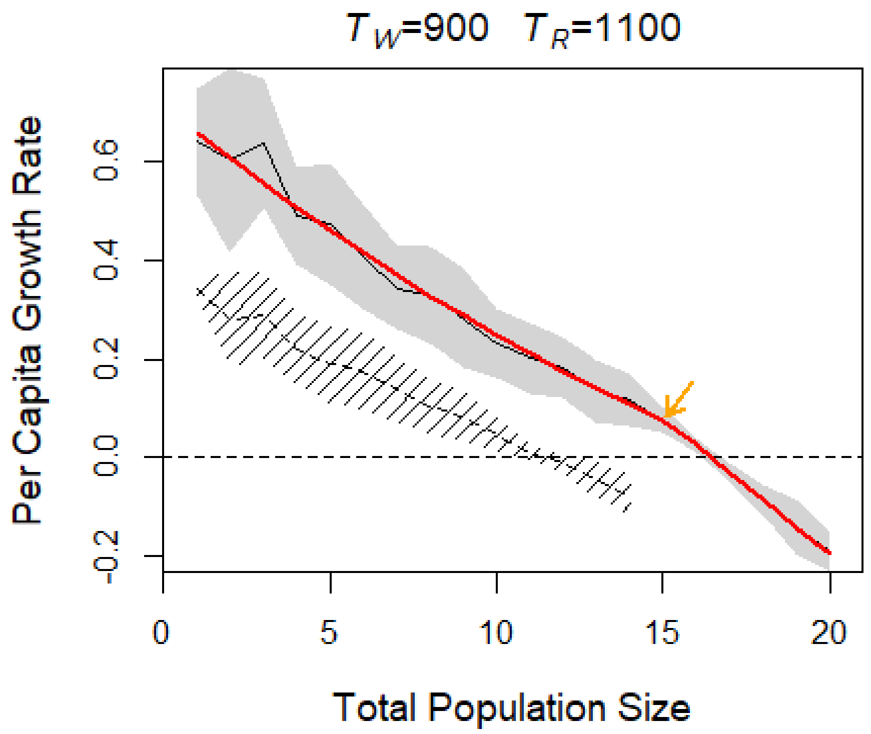

T_W=900 T_R=1100
Per Capita Growth Rate
-0.2
0.0
0.2
0.4
0.6
0
5
10
15
20
Total Population Size

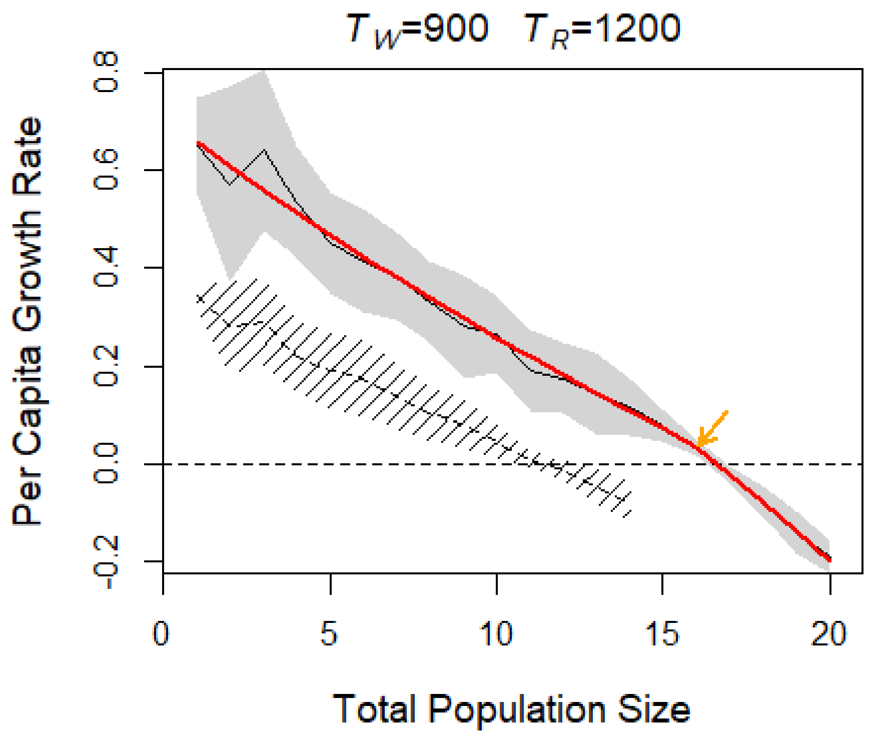

T_W=900 T_R=1200
Per Capita Growth Rate
-0.2
0.0
0.2
0.4
0.6
0.8
0
5
10
15
20
Total Population Size

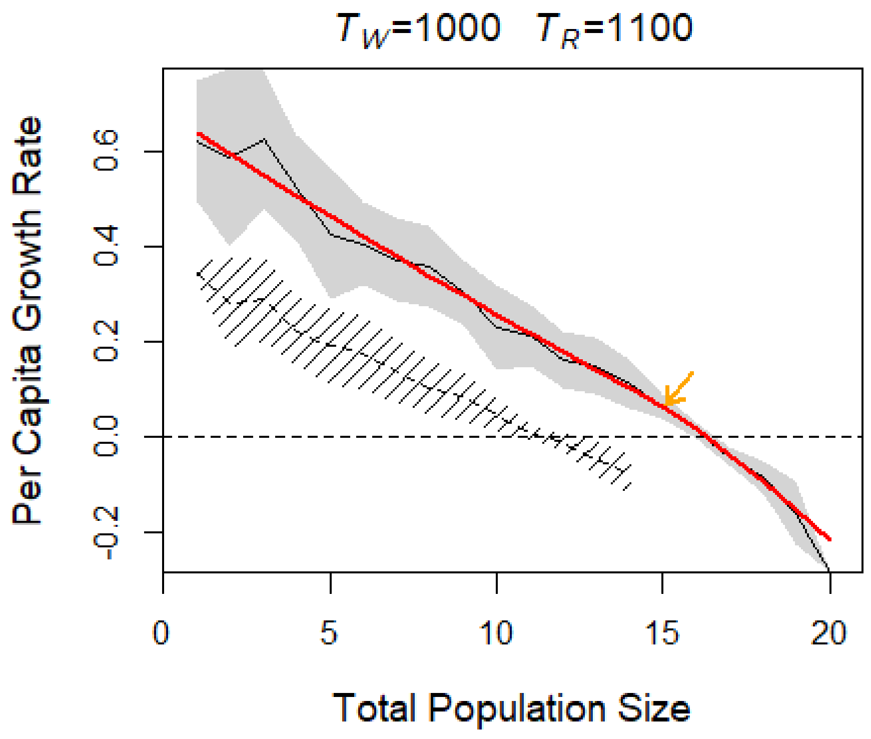

T_W=1000 T_R=1100
Per Capita Growth Rate
-0.2
0.0
0.2
0.4
0.6
0
5
10
15
20
Total Population Size

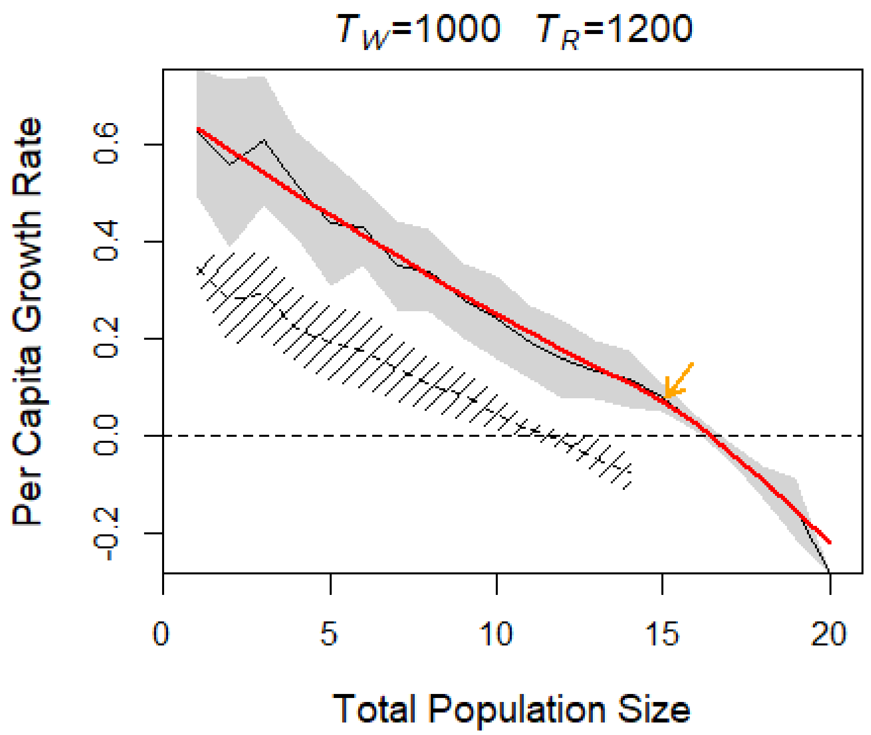

Fig. S9. Mean + SD (between simulations) per capita growth rate as a function of total population size for populations of with-memory individuals (plain line and grey area). All parameters were set to default values, except T_W and T_R. (dashed line and zebra area correspond to populations of memoryless individuals shown here only for comparative purpose). When the estimation procedure of the piecewise 2nd-order polynomial regression (with-breakpoint model) converges, the predicted values are represented by a red line, and the location of the estimated breakpoint by an orange arrow.

Supporting Information 3

Sensitivity of the carrying capacity and the shape of the density-dependence of populations of with-memory individuals to the attractiveness threshold used by individuals when determining valuable patches.

The carrying capacity is robust to variations in the attractiveness threshold used by individuals to determine valuable patches V_{thr}, with a mean value of 14.1 ± 1.4 and 16.0 ± 0.2 for V_{thr} = 0.005 and 0.02 respectively. For V_{thr} =0.005 (all other parameters set to default values), we could detect breakpoint (ΔAIC > 100 with the simple 2nd-order polynomial regression, the Beverton-Holt and the Ricker models, ΔAIC > 50 with the theta-logistic model, see Fig. S10, left panel: predicted values in red, estimated breakpoint indicated by the orange arrow) that occurs at an estimated mean population size of 13.1. At this population size, the slope of the density-dependence changes from positive (0.01) to negative (-0.04). For V_{thr}=0.02, no breakpoint occurs and the piecewise 2nd-order polynomial model does not converge. The best-fitting model is then a theta-logistic model with theta estimated as 1.7 ± 0.1 (estimated + SE, all ΔAIC > 2 see Fig. S10, right panel).

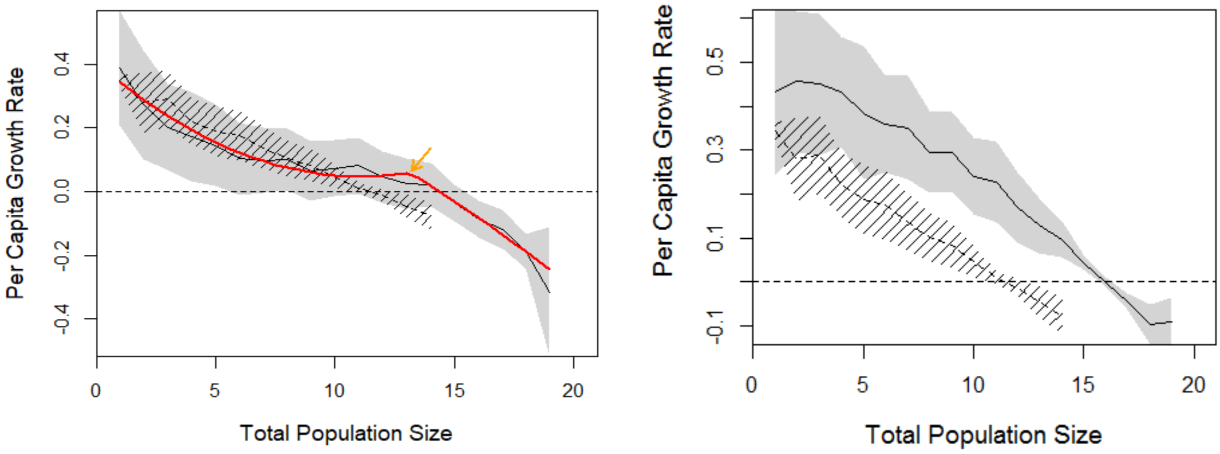

Fig. S10. Mean ± SD (between simulations) per capita growth rate as a function of total population size for populations of with-memory individuals (plain line and grey area). All parameters were set to default values, except V_{thr}, which was set to 0.005 (left panel) and 0.02 (right panel) (dashed line and zebra area correspond to populations of memoryless individuals shown here only for comparative purpose).

Note that a small V_{thr} value (left panel) leads to smaller per capita growth rates because with-memory individuals go back to poor or distant known patches more frequently than when the threshold is larger, thus decreasing their foraging efficiency. On the other hand, as there is a range of V_{thr} values for which individuals can always, after a learning phase, improve their intake rate (cf Riotte-Lambert et al. 2015[2]), increasing V_{thr} within this range always lengthens the learning phase, but also improves the performance of individuals on the long run because they use only the best patches. This explains why for a larger V_{thr} the population dynamics of with-memory individuals diverges even more from that of populations of memoryless individuals (right panel).

---

[2] Riotte-Lambert, L., Benhamou, S. and S. Chamaillé-Jammes. 2015. How memory-based movement leads to nonterritorial spatial segregation. *The American Naturalist*. 185: E103-E116

Supporting Information 4

Sensitivity of the carrying capacity and of the shape of the density-dependence to the energetic cost of movement.

With all other parameters set to default values, for an energetic cost of movement equal to twice the default value, 100% of populations of without-memory individuals and 76% of populations of with-memory individuals go extinct. The remaining populations of this latter type do not grow and stay at a mean population size of 1.96 (± 1). For an energetic cost of movement equal to half the default value, the mean ± SD carrying capacity increases to 25.78 ± 0.4 (with memory) and 25.07 ± 0.54 (without memory). At the end of the simulation, the mean ± SD intake rate is similar for with- and without-memory individuals (2.32 ± 0.17 vs 2.23 ± 0.10).

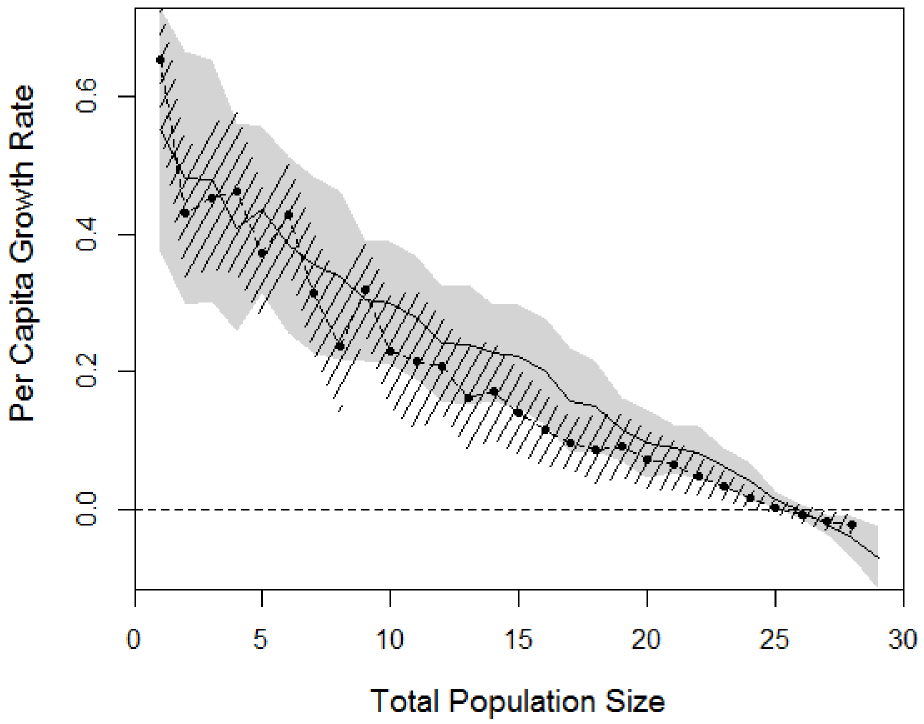

Fig. S11. Mean ± SD (between simulations) per capita growth rate as a function of the total population size, for both types of populations (with-memory: plain line and grey area; without memory: dashed line and zebra area). All parameters were set to default values, except the energetic cost of movement, which was set to 0.025, i.e. half the default value. No breakpoint is present in this case.

Fig. S12 shows two examples of the spatial patterns for the last time window of the simulation of a population of with-memory individuals with a cost of movement set to default value vs half the default value (left and right panels). It appears that individuals are more numerous and each uses larger areas when the cost of movement is low, which leads to an increased overlap.

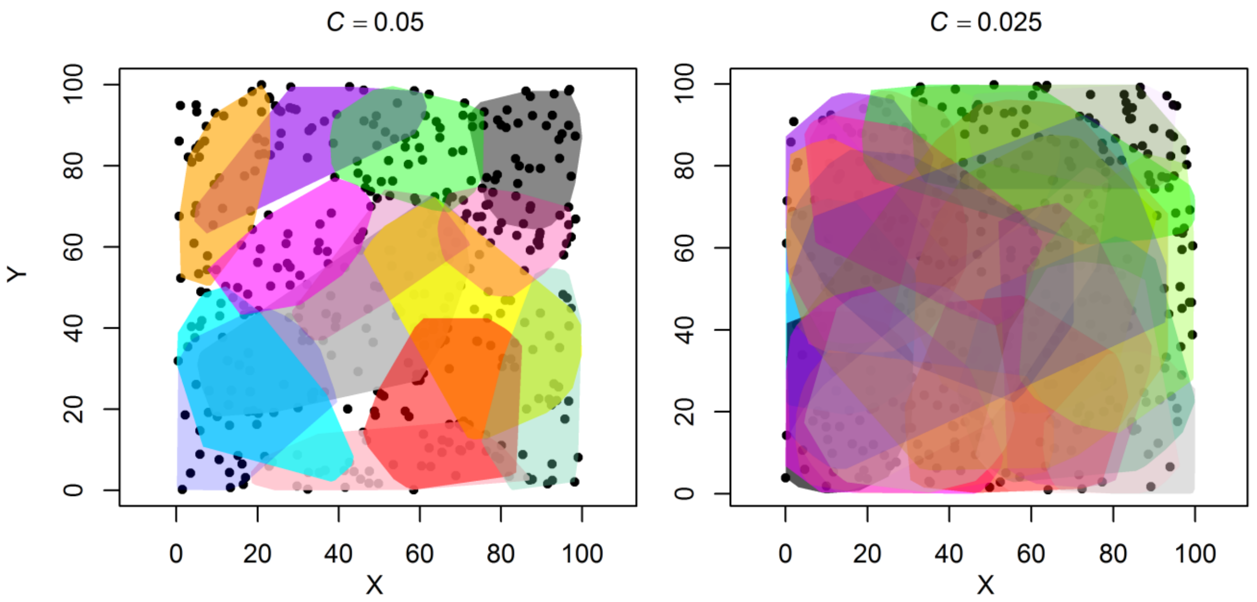

Fig. S12. Individuals' ranges are represented by 95% Maximum Convex Polygon drawn with different colours. Resource patches are represented by black dots. The population size reaches 14 individuals for *c* = 0.05 (left panel) and 25 for c = 0.025 (right panel).

Supporting Information 5

Sensitivity of the carrying capacity and the shape of the density-dependence to the value of the energetic threshold that an individual must reach to reproduce.

The carrying capacity of the two types of populations is robust to changes in the difference between the energetical state E_{rep} that an individual must reach to reproduce and the value to which its state is lowered following a reproduction event (Fig. S13). The carrying capacity is equal to 16.67 $\pm$ 0.19 (with memory) and 11.45 $\pm$ 0.41 (without memory) for a halved difference (E_{rep}=375), and is equal to 16.11 $\pm$ 0.35 (with memory) and 11.20 $\pm$ 0.50 (without memory) for a doubled difference (E_{rep}=750).

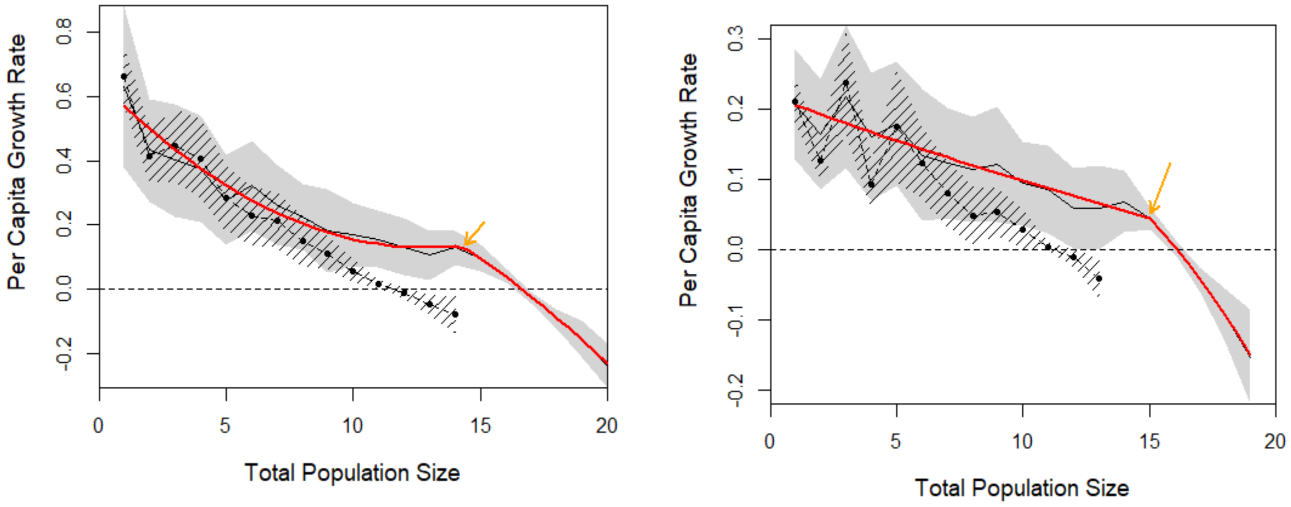

Fig. S13. Mean $\pm$ SD (between simulations) per capita growth rate as a function of total population size for both types of populations (with-memory: plain line and grey area; without memory: dashed line and zebra area). All parameters were set to default values, except E_{rep}, which was set to 375 (left panel) and 750 (right panel). The red line represents the predicted values of the piecewise 2nd-order polynomial regression fitted to populations of memory users. The estimated location of the breakpoint is indicated by an orange arrow.

For E_{rep}=375, the shape of density-dependence for populations of with-memory individuals shows a breakpoint (all ΔAIC > 100 with the no-breakpoint 2nd-order polynomial, the Beverton-Holt, Ricker and theta-logistic models) estimated at a population size of 14. On the left side of this point**,** the slope of density-dependence is slightly positive (0.008), and on the right side, the slope abruptly becomes negative (-0.05). For E_{rep}=750, a breakpoint is also present (all ΔAIC > 80) and occurs at an estimated population size of 15. At this population size, the slope of density-dependence is 3.7 times greater. Note that for E_{rep}=750, we ran the simulations for 400,000 time steps instead of 200,000 to allow for the population to reach its carrying capacity.

Supporting Information 6

Comparison of three phenomenological models
of density-dependence fitted on r-N curves

Populations of with-memory individuals

Fig. S14 shows the per capita growth rate as a function of total population size for populations of with-memory individuals, along with the values predicted by various models. The model parameters are given in Table S1.

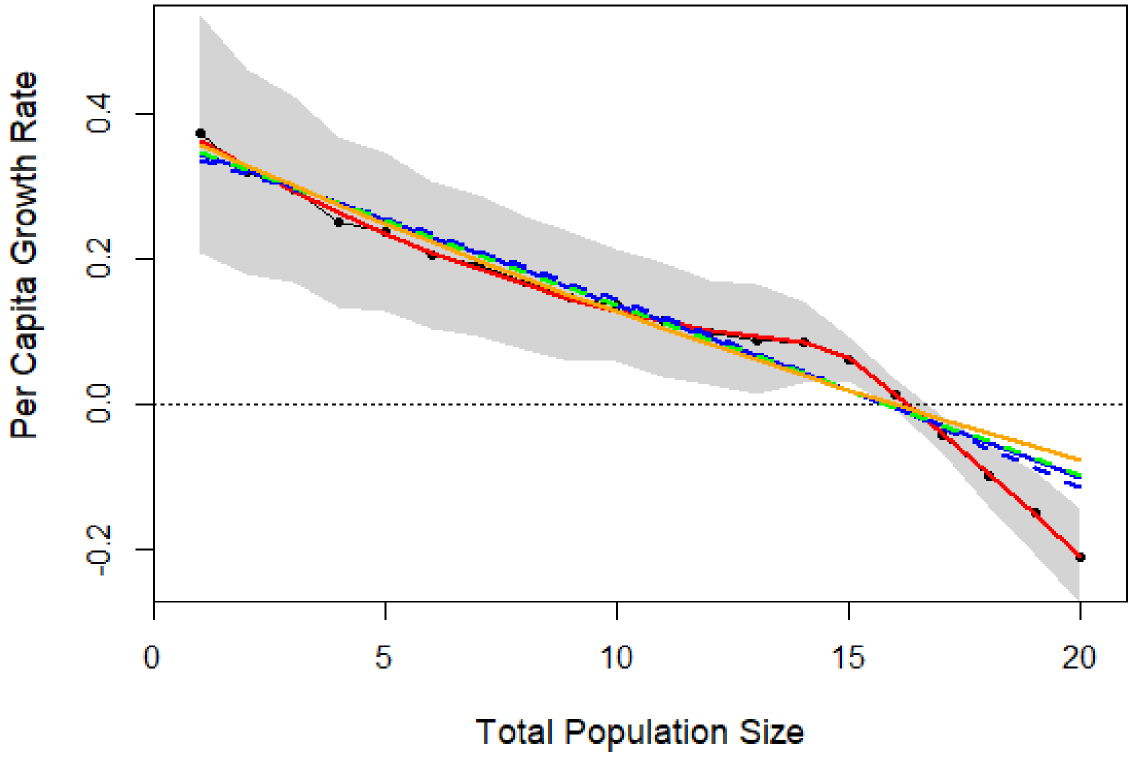

Fig. S14. Mean ± SD (between simulations) per capita growth rate as a function of total population size for populations of with-memory individuals (plain line and grey area). The various models we attempted to fit are a 2nd-order polynomial regression (dashed blue line), a piecewise 2nd-order polynomial regression (red line), the Beverton-Holt (orange line), Ricker (dashed green line) and theta-logistic (plain blue line) models.

Table S1: Model AIC and parameter estimates for populations of with-memory individuals

Model name	AIC	formula	Estimated parameters ± SE
Piecewise 2nd-order polynomial regression	-26921	$r \sim (n<=BP)*(\alpha + a*n + b*n^2)$ $+ (n>BP)*(\alpha + (a-a_2)*BP +$ $(b-b_2)*(BP^2) + a_2*n + b_2*n^2)$	BP = 15 ± 0<br>α = 0.4 ± 0.0<br>a = -0.04 ± 0.00<br>b = 0.001 ± 0.000<br>a_2 = -0.02 ± 0.03<br>b_2 = -0.0009 ± 0.0009
2nd-order polynomial regression	-25894	$r \sim \alpha + a*n + b*n^2$	α = 0.36 ± 0.00<br>a = -0.02 ± 0.00<br>b = -0.0002 ± 0.0000
Theta-logistic	-25847	$r \sim R_{max}*(1 - (n/K)^{\theta})$	θ = 1.04 ± 0.02<br>R_{max} = 0.37 ± 0.00<br>K = 16 ± 0
Linear (Ricker)	-25847	$r \sim \alpha + a*n$	α = 0.37 ± 0.00<br>a = -0.02 ± 0.00
Beverton-Holt	-25692	$r \sim R_{max} - \ln[1 + (e^{Rmax} -1)*n/K]$	R_{max} = 0.39 ± 0.00<br>K = 16 ± 0

Populations of memoryless individuals

Fig. S15 shows the per capita growth rate as a function of total population size for populations of without-memory individuals, along with the values predicted by various models. The model parameters are given in Table S2.

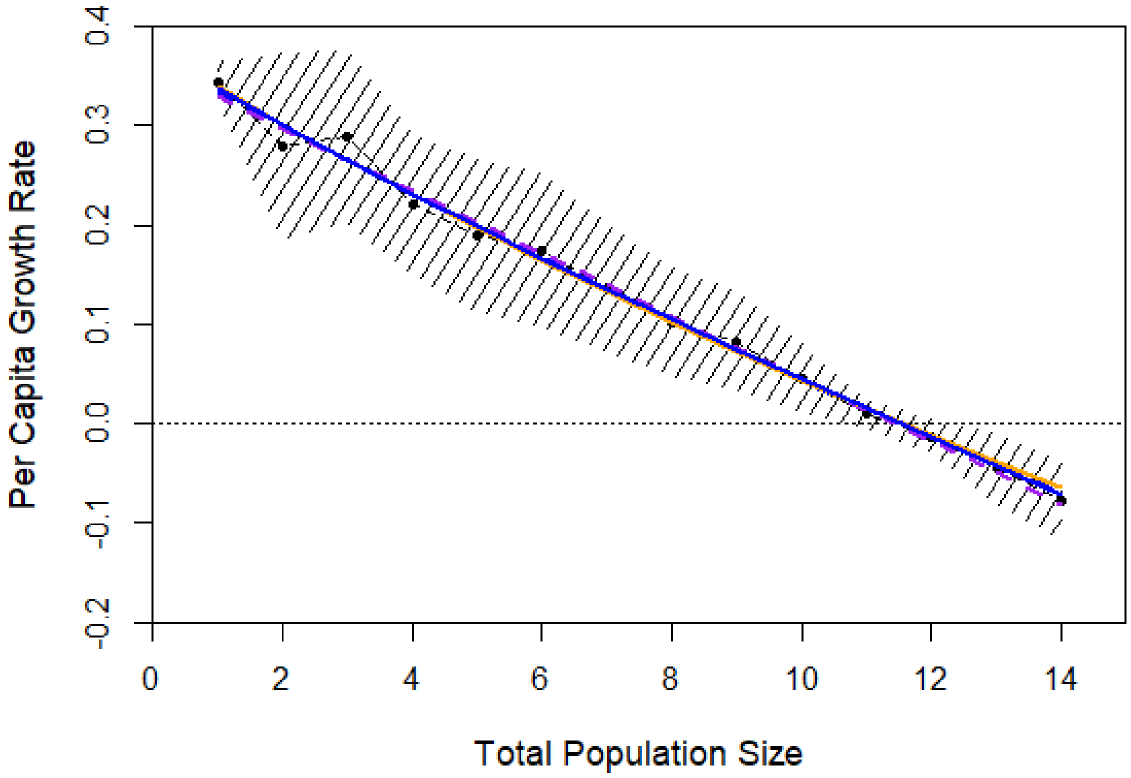

Fig. S15. Mean ± SD (between simulations) per capita growth rate as a function of total population size for populations of memoryless individuals (dashed line and zebra area). The various models we attempted to fit are a 2nd-order polynomial regression (dashed blue line), a piecewise 2nd-order polynomial regression (red line), and the Beverton-Holt (orange line), theta-logistic (plain blue line), and Ricker (i.e. linear) (dashed purple line) models. The fitting procedure of a piecewise 2nd-order polynomial regression did not converge.

Table S2: Model AIC and parameter estimates for populations of without-memory individuals

Model name	AIC	formula	Estimated parameters $\pm$ SE
Theta-logistic	-30055	$r \sim R_{max}*(1 – (n/K)^{\theta})$	$\theta = 0.88 \pm 0.02$<br>$R_{max} = 0.38 \pm 0.00$<br>$K = 11.5 \pm 0.0$
2nd-order polynomial regression	-30044	$r \sim \alpha + a*n + b*n^2$	$\alpha = 0.37 \pm 0.00$<br>$a = -0.035 \pm 0.001$<br>$b = 0.00029 \pm 0.00005$
Beverton-Holt	-30033	$r \sim R_{max} – \ln[1 + (e^{Rmax} -1)*n/K]$	$R_{max} = 0.38 \pm 0.00$<br>$K = 12 \pm 0$
Linear (Ricker)	-30010	$r \sim \alpha + a*n$	$\alpha = 0.36 \pm 0.00$<br>$a = -0.03 \pm 0.00$
